# Nine viruses from eight lineages exhibiting new evolutionary modes that co-infect a hypovirulent phytopathogenic fungus

**DOI:** 10.1371/journal.ppat.1009823

**Published:** 2021-08-24

**Authors:** Fan Mu, Bo Li, Shufen Cheng, Jichun Jia, Daohong Jiang, Yanping Fu, Jiasen Cheng, Yang Lin, Tao Chen, Jiatao Xie

**Affiliations:** 1 State Key Laboratory of Agricultural Microbiology, Huazhong Agricultural University, Wuhan, China; 2 Hubei Hongshan Laboratory, Wuhan, China; 3 Hubei Key Laboratory of Plant Pathology, College of Plant Science and Technology, Huazhong Agricultural University, Wuhan, China; Okayama University, JAPAN

## Abstract

Mycoviruses are an important component of the virosphere, but our current knowledge of their genome organization diversity and evolution remains rudimentary. In this study, the mycovirus composition in a hypovirulent strain of *Sclerotinia sclerotiorum* was molecularly characterized. Nine mycoviruses were identified and assigned into eight potential families. Of them, six were close relatives of known mycoviruses, while the other three had unique genome organizations and evolutionary positions. A deltaflexivirus with a tripartite genome has evolved via arrangement and horizontal gene transfer events, which could be an evolutionary connection from unsegmented to segmented RNA viruses. Two mycoviruses had acquired a second helicase gene by two different evolutionary mechanisms. A rhabdovirus representing an independent viral evolutionary branch was the first to be confirmed to occur naturally in fungi. The major hypovirulence-associated factor, an endornavirus, was finally corroborated. Our study expands the diversity of mycoviruses and potential virocontrol agents, and also provides new insights into virus evolutionary modes including virus genome segmentation.

## Author summary

Identification of mycoviruses in phytopathogenic fungi is necessary for understanding the origin of viruses and developing virocontrol strategies to protect plants. Nine mycoviruses with RNA genomes were identified in a hypovirulent strain of *Sclerotinia sclerotiorum* and were classified into eight potential viral families, suggesting that the composition of mycoviral communities was complex in this single fungal strain. They included four previously characterized mycoviruses and three distant relatives of known mycoviruses, as well as the first reports of a deltaflexivirus with a tripartite genome, and a fungal rhabdovirus. In addition, we found an endornavirus associated with hypovirulence in a phytopathogenic fungus. Our study makes a significant contribution because it not only expands the diversity-related knowledge of mycoviruses and potential virocontrol agents, but also provides new insights into mycovirus evolution.

## Introduction

Mycoviruses (or fungal viruses), an important component of the virosphere, are prevalent in major fungal kingdom groups, and have a vast genetic diversity with either RNA or DNA genomes [1]. Mycoviruses have been assigned to 26 known families (https://talk.ictvonline.org/ictv-reports/ictv_online_report/). Recently, an increasing number of mycoviruses representing novel viruses with unique genomes or evolutionary positions, but broadly referred to as unclassified, have been discovered and characterized [2–8]. The discovery of more mycoviruses will expand our knowledge regarding virus diversity, classification, and evolution. Although most known mycovirus infections do not trigger a noticeable reaction, a few mycoviruses have a demonstrated role in hypovirulence/hypervirulence or other complex phenotypic changes in their fungal hosts. Moreover, mycoviruses related to hypovirulence highlight their possible use as disease control agents in agriculture [1].

Viral co-infections are a common phenomenon in nature. Viral co-infections could supply a chance for virus communication via recombination or horizontal gene transfer, which may promote the evolution of viruses and allow the spread of unique features to adapt to environmental changes [9,10]. The advanced techniques of next-generation sequencing have confirmed that co-infection with multiple mycoviruses is unexpectedly complex and diverse in a single fungal strain [11–13], and have enabled exploration of viral evolution from a different perspective. Interactions between mycoviruses may enhance or decrease viral disease symptoms via synergistic or antagonistic mechanisms [14,15]. The spread of mycoviruses is usually considered to be limited by the vegetative incompatibility of fungal isolates. However, it is noteworthy that a single strain co-infected with multiple related or/and distant mycoviruses was isolated with high frequency. For instance, mycoviruses have been molecularly characterized in 23 *Sclerotinia sclerotiorum* strains, and more than 80% (19/23) were co-infected by multiple related or/and distinct mycoviruses [16]. The complexity of co-infections enables the virus population to spread systemically and successfully survive under natural conditions, perhaps by the complementing functions of different viruses that facilitate adaptation to new environments. A mycoreovirus, Sclerotinia sclerotiorum mycoreovirus 4, can suppress fungal non-self recognition reactions and facilitate horizontal transmission of heterologous mycoviruses [17]. A *Rosellinia necatrix* strain was co-infected by a capsidless (+)ssRNA virus (yado-kari virus, YkV1) and a particulate dsRNA virus (yado-nushi virus, YnV1), where YkV1 could hijack the virions of YnV1 to protect itself [18].

Nonsegmented and segmented (-)ssRNA mycoviruses have been routinely discovered in fungi, and assigned into diverse orders including *Mononegavirales*, *Jingchuvirales*, *Serpentovirales*, *Muvirales*, *Goujianvirales*, *Bunyavirales* and *Articulavirales*, and some of them routinely discovered in fungi. Rhabdoviruses are widespread in animals and plants. However, no member within *Rhabdoviridae* has been characterized in fungi. Typical virions of rhabdoviruses are characteristic bullet-shaped or cone-shaped in animal hosts [19] and bacilliform in plant hosts [20,21] with genomes of approximately 10–16 kb. The genomic organization of members within the *Rhabdoviridae* usually includes five canonical open reading frames (ORFs) that encode (from 3’ to 5’) the nucleoprotein (N), phosphoprotein (P), matrix protein (M), glycoprotein (G), and large protein (L, RNA-dependent RNA polymerase) [19,22].

*S*. *sclerotiorum* is a destructive necrotrophic fungal pathogen in the Ascomycota and causes diseases of Sclerotinia stem rot. This pathogen has the widest-known host range and can infect more than 700 plant species, including important crops and numerous weeds [23]. Recently, *S*. *sclerotiorum* was found to be an endophytic fungus in cereal crops and beneficial to resistance against major pathogens [24]. *S*. *sclerotiorum* harbors rich diverse mycoviruses including dsRNA, ssRNA, and DNA mycoviruses, of which ssRNA mycoviruses are dominant [25,26]. Some of those identified mycoviruses were positioned in a critical evolutionary node, contributing to knowledge on viral evolutionary history [5,27–29]. Some mycoviruses associated with hypovirulence have been demonstrated to be resources for Sclerotinia disease management [30,31]. Two endornaviruses ((+)ssRNA viruses), Sclerotinia sclerotiorum endornavirus 1 and 2 (SsEV1 and SsEV2), have been reported in *S*. *sclerotiorum* with symptomless infections [32,33]. Endornaviruses are common infections in filamentous fungi, but only two endornaviruses, Helicobasidium mompa endornavirus 1 (HmEV1) and Sclerotinia minor endornavirus 1 (SmEV1), have been associated with the hypovirulence [34,35].

In this study, we molecularly characterized nine mycoviruses infecting the hypovirulent strain SX276 of *S*. *sclerotiorum*. Three of nine mycoviruses showed unique genome organization and evolutionary position. A deltaflexivirus with a tripartite (+)ssRNA genome has evolved via arrangement and horizontal gene transfer events, and provides an evolutionary link between segmented and non-segmented viruses. Two viruses including deltaflexivirus harbor two helicase genes in their genomes which reveal different evolutionary mechanisms. A rhabdovirus belongs to the subfamily *Alpharhabdovirinae* was the first confirmation of a natural occurrence in fungi, and was defined to represent an independent viral evolutionary branch. An endornavirus was associated with the hypovirulence of *S*. *sclerotiorum* strain SX276. Our research has enriched viral diversity in terms of genomic organization and provided new insights into evolution and divergence.

## Results

### *S*. *sclerotiorum* strain SX276 harboring multiple dsRNA species shows hypovirulence

**Fig 1:**
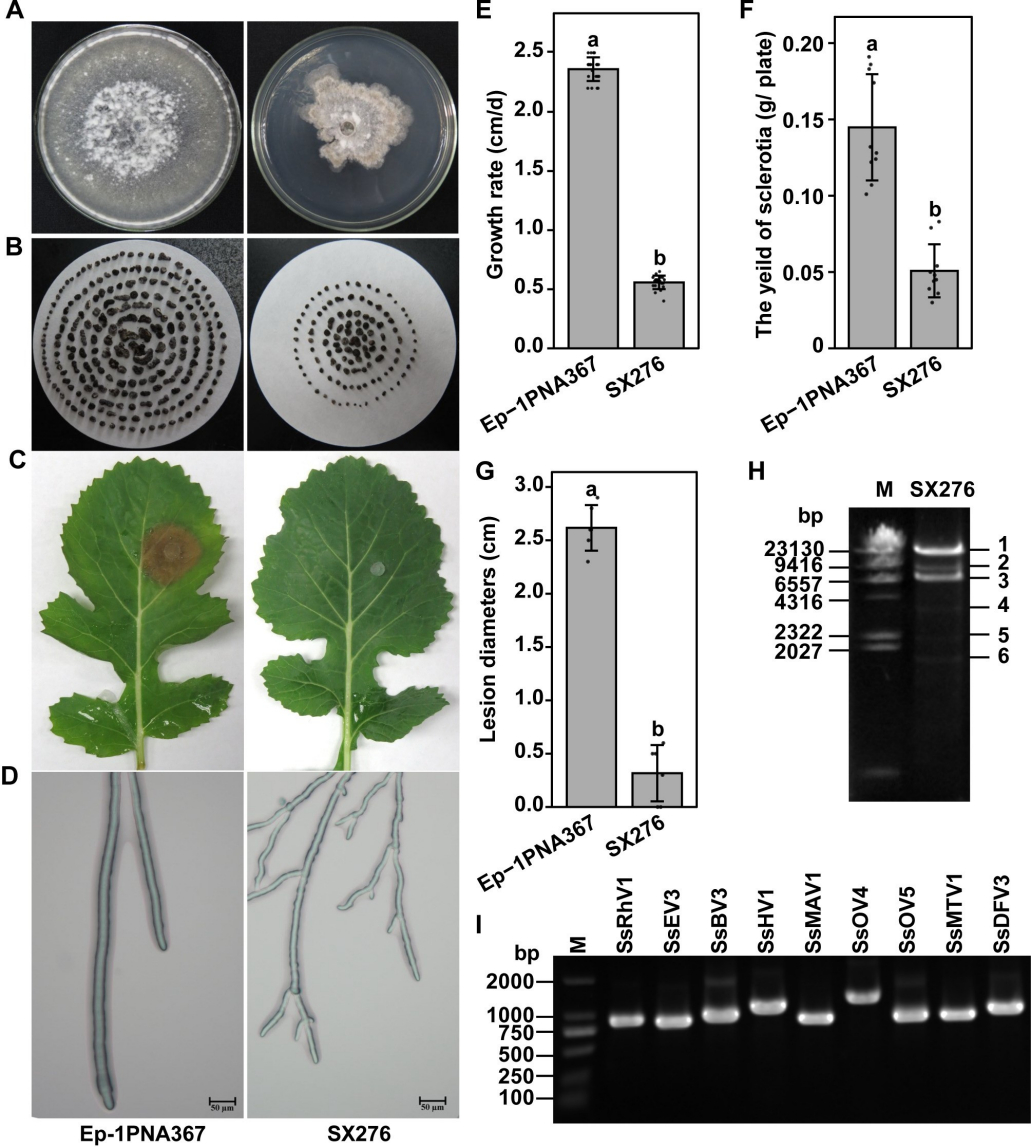
*S*. *sclerotiorum* strain SX276 shows hypovirulent phenotypes. (A) Colony morphology of strains Ep-1PNA367 and SX276. All strains were cultured on PDA plates for 7 days prior to photography. (B) Sclerotia of strains Ep-1PNA367 and SX276. Sclerotia from ten PDA plates of each strain were collected and photographed together. (C) Pathogenicity assay of strains Ep-1PNA367 and SX276 were carried out on detached leaves. Photos and data were taken at 48 hours post-inoculation. (D) Hyphal tips of strains Ep-1PNA367 and SX276. (E) Growth rate of strains Ep-1PNA367 and SX276 under laboratory conditions at 20°C. (F) The yield of strains Ep-1PNA367 and SX276. (G) Lesion diameters induced by the two strains on detached rapeseed leaves (20°C, 48 hours post-inoculation). (H) Agarose gel electrophoresis of the dsRNA elements associated with strain SX276. (I) RT-PCR confirmation of *de novo* assembled contigs from strain SX276 generated by Illumina sequencing. Lane M: DL2000 DNA molecular weight Marker (Takara Dalian, China); lane 1 to 9: abbreviations of viruses (see Table 1 for detail). Error bars indicate the SD from six sample means. The different letters on the top of each column indicate significantly difference at the P < 0.05 level of confidence according to the *t* test.

Compared to normal strain Ep-1PNA367 (the virulent isolate), strain SX276 showed a reduced growth rate and abnormal colony morphology with the significantly smaller and lower number of sclerotia on potato dextrose agar (PDA) (Fig 1A, 1B, 1E and 1F). The strain Ep-1PNA367 caused typical lesions on detached leaves of rapeseed, whereas strain SX276 failed to infect the leaves (Fig 1C and 1G). Moreover, compared to Ep-1PNA367, the hyphal tips of strain SX276 curved and were found to extend predominantly through branching (Fig 1D). These results suggested that strain SX276 is a typical hypovirulent strain.

The dsRNA species were successfully extracted from the strain SX276. The results of the gel electrophoresis demonstrated that the strain SX276 possesses at least six distinct dsRNA segments ranging between 1.6 and 23 kbp in size (Fig 1H), which suggests that it bears a diverse pattern of dsRNA mycoviruses and/or replicative intermediates of ssRNA mycoviruses.

### Nine mycoviruses co-infecting a single strain shows remarkable diversity

To investigate the mycoviruses infecting strain SX276, the metatranscriptome was sequenced and analyzed. The Illumina sequencing generated more than 13 Gb of raw reads, and after trimming the low-quality reads, the sample contained 12.08 Gb high-quality sequence reads. The clean reads were *de novo* assembled into 16,684 large contigs. Some contigs (11%, 1,870/16,684) could not be annotated, but 84% (13,986/16,684) matched the *S*. *sclerotioru*m genome, and 5% (828/16,684) were related to viruses.

Among the virus-related contigs, there were 192 contigs of more than 1000 nt and these contigs were compared to remove sequences that had more than 90% similarity with each other. The remaining contigs were subjected to BLASTx analysis against the GenBank/ENA/DDBJ nr database. The assembled contigs represented partial sequences of nine different putative mycoviruses, including seven (+)ssRNA viruses, one dsRNA virus, and one (-)ssRNA virus (Table 1). To identify these nine putative mycoviruses in the strain SX276, reverse-transcription PCR (RT-PCR) was performed using virus-specific primers (S1 Table). The predicted bands were amplified from the cDNA library of the total RNA from strain SX276 (Fig 1I).

**Table 1:** The information of the nine viruses in strain SX276.

Virus name	Accession No.	Length (nt)	Best match (BLASTx)	Identities	Query cover	E values	Reads numbers	Reads number/kb	Genome Type	Family/Genus
Sclerotinia sclerotiorum rhabdovirus 1 (SsRhV1)	MT706019	11356	Piry virus	37%	48%	0	65231	5744.19	(-)ssRNA	*Rhabdoviridae*
Sclerotinia sclerotiorum botybirnavirus 3 S1 (SsBV3S1)	MT829323	6214	Sclerotinia sclerotiorum botybirnavirus 3	98%	91%	0	7920611	1274639.68	dsRNA	*Botybirnavirus*
Sclerotinia sclerotiorum botybirnavirus 3 S2 (SsBV3S2)	MT829324	5880	Sclerotinia sclerotiorum botybirnavirus 3	99%	91%	0	7876512	1339542.86		
Sclerotinia sclerotiorum endornavirus 3 (SsEV3)	MT706020	12631	Sclerotinia sclerotiorum endornavirus 1	27%	63%	0	349115	27639.54	(+)ssRNA	*Endornaviridae*
Sclerotinia sclerotiorum hypovirus 1 (SsHV1)	MT829322	10430	Sclerotinia sclerotiorum hypovirus 1	97%	85%	0	185561	17791.08	(+)ssRNA	*Hypoviridae*
Sclerotinia sclerotiorum mycotymovirus 1 (SsMTV1)	MT706023	7098	Sclerotinia sclerotiorum mycotymovirus 1	97%	82%	0	15524	2187.09	(+)ssRNA	*Tymoviridae*
Sclerotinia sclerotiorum ourmia-like virus 4 (SsOV4)	MT706021	2905	Sclerotinia sclerotiorum ourmia-like virus 4	97%	82%	0	1450	499.14	(+)ssRNA	*Botourmiaviridae*
Sclerotinia sclerotiorum deltaflexivirus 3 S1 (SsDFV3S1)	MT706024	7403	Fusarium graminearum deltaflexivirus 1	42%	68%	0	135626	18320.41	(+)ssRNA	*Deltaflexiviridae*
Sclerotinia sclerotiorum deltaflexivirus 3 S2 (SsDFV3S2)	MW452524	2560	Sclerotinia sclerotiorum deltaflexivirus 1	31%	18%	0	44288	17300		
Sclerotinia sclerotiorum deltaflexivirus 3 S3 (SsDFV3S3)	MW452525	1827	Sclerotium rolfsii alphavirus-like virus 3	38%	42%	0.01	78110	42753		
Sclerotinia sclerotiorum ourmia-like virus 5(SsOV5)	MT706022	2823	Sclerotinia sclerotiorum ourmia-like virus 2	44%	66%	2.00 E-155	877	310.66	(+)ssRNA	*Botourmiaviridae*
Sclerotinia sclerotiorum mycoalphavirus virus 1(SsMAV1)	MT706025	7531	Sclerotinia sclerotiorum RNA virus L	35%	36%	0	91711	12177.80	(+)ssRNA	Unclassified

The terminal sequences of individual mycoviruses were obtained via a modified RACE (rapid amplification of cDNA ends). Combined with the information from all the results mentioned above, the full genomes of nine mycoviruses were assembled, and their detailed information is shown in Table 1. In summary, strain SX276 was unequivocally confirmed to be co-infected by nine different mycoviruses. Based on their genome characterizations, those nine mycoviruses temporarily were designated as Sclerotinia sclerotiorum hypovirus 1 (SsHV1/SX276), Sclerotinia sclerotiorum botybirnavirus 3 (SsBV3/SX276), Sclerotinia sclerotiorum ourmia-like virus 4 (SsOV4/SX276), Sclerotinia sclerotiorum ourmia-like virus 5 (SsOV5/SX276), Sclerotinia sclerotiorum deltaflexivirus 3 (SsDFV3/SX276), Sclerotinia sclerotiorum endornavirus 3 (SsEV3/SX276), Sclerotinia sclerotiorum mycotymovirus 1 (SsMTV1/SX276), Sclerotinia sclerotiorum mycoalphavirus virus 1 (SsMAV1/SX276), and Sclerotinia sclerotiorum rhabdovirus 1 (SsRhV1/SX276). Among the identified mycoviruses, SsHV1/SX276, SsBV3/SX276, SsMTV1/SX276, and SsOV4/SX276 shared high identities (>97%) with previously reported viruses (Table 1). An ourmia-like virus, SsOV5, consisted of a single larger ORF encoding a putative RNA-directed RNA polymerase (RdRP) (S1 Fig), and showed 44% identity with Sclerotinia sclerotiorum ourmia-like virus 2 (KP900929) (Table 1). In the following research, we focused on four mycoviruses because of their unique evolutionary or biological features.

### A deltaflexivirus contains three RNA segments derived from its unsegmented relatives and heterologous viruses

**Fig 2:**
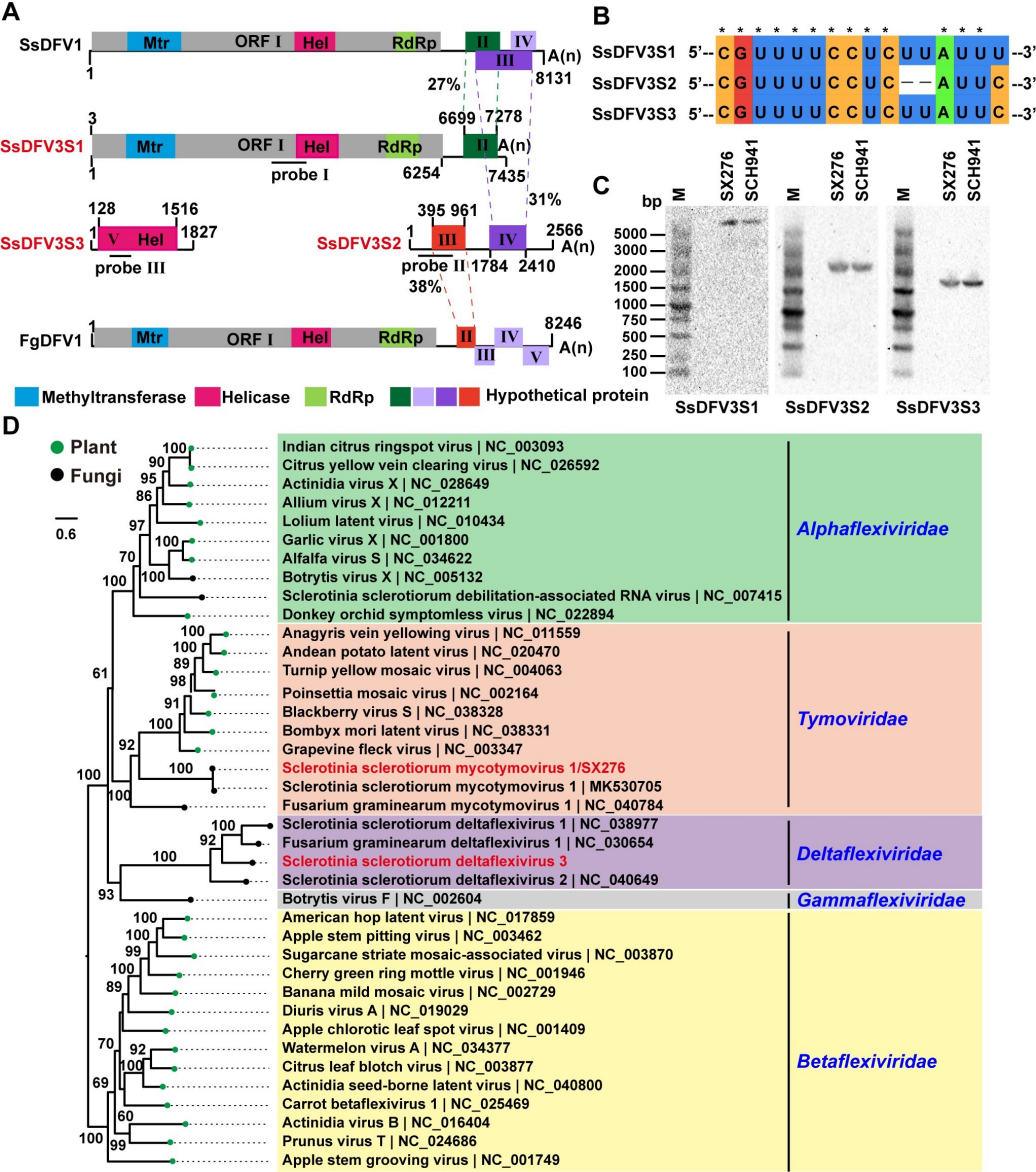
Genome organization and maximum likelihood tree of SsMTV1/SX276 and SsDFV3. (A) The organizations of SsDFV3 and other viruses in family *Deltaflexiviridae*. Open reading frames (ORFs) are shown as boxes. (B) Multiple alignments of 5′-terminal nucleotide sequences of the SsDFV3 genomic segments. (C) Northern blot of three segments of SsDFV3 in strain SX276 and SCH941, the probes are marked with a horizontal line in Fig 2A. (D) Maximum likelihood tree of SsMTV1/SX276 and SsDFV3 was constructed based on amino acid alignments of RdRp.

SsDFV3 has a tripartite genome and contains five ORFs (ORF I to ORF V) (Fig 2A). Three RNA segments (SsDFV3S1-S3) always co-exist in the same fungal isolate and share a conserved 5′-terminal sequence (5′-CGUUUUCCUC-3′), indicating that the three RNA segments were associated each other and formed a single SsDFV3 genome (Figs 2B and S2A). The northern blot of the SsDFV3S1-S3 was performed, and no subgenomic RNAs are found (Fig 2C). SsDFV3S1 is composed of 7,435 nt in length and has two putative ORFs. ORF I encodes a 2,070 aa polyprotein with three conserved domains of methyltransferase (Mtr), helicase (Hel; helicase 1, He1-1), and RdRP, that shared 39% identity to the replication-associated polyprotein of Fusarium graminearum deltaflexivirus 1 (FgDFV1, KX015962) (Fig 2A and S2 Table). ORF II putatively encodes a protein with an unknown function, which has a 27% identity to the protein encoded by ORF II of SsDFV1(S2 Table). SsDFV3S2 contains 2,566 nt length and has two ORFs (ORF III and ORF IV). The protein encoded by ORF III has a 39% identity to the hypothetical protein encoded by ORF II of FgDFV1, while ORF IV encodes a hypothetical protein that shared 39% identity to that encoded by ORF III of SsDFV1 (S2 Table). The smallest segment, SsDFV3S3, has a single ORF (ORF V) and putatively encodes a protein containing the second helicase domain (helicase 2, Hel-2) that has 27% identity to the helicase of Sclerotium rolfsii alphavirus-like virus 3 (AZF86095) (Fig 2A and S2 Table). To predict the function of helicase 2 encoded by SsDFV3S3, alignment by hidden Markov model (HMM) matching using HHpred (https://toolkit.tuebingen.mpg.de/tools/hhpred) was performed. The result indicated that the helicase 2 showed 99% confidence with the triple gene block 1 (TGB1) from plant viruses (S3 Fig), but whether the helicase2 has specific biological functions same with TGB1 need to be explored in future research.

**Fig 3:**
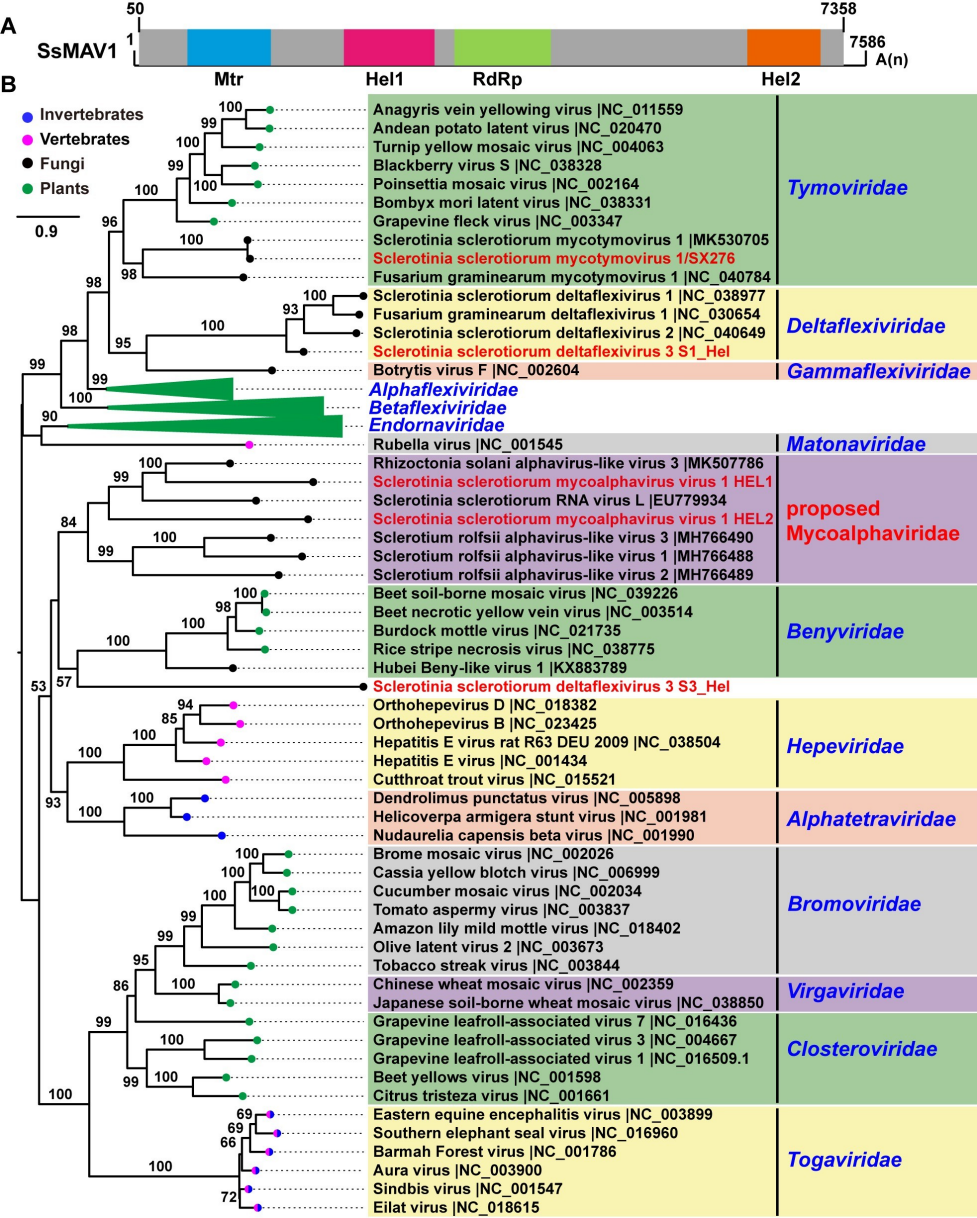
Genome organization of SsMAV1 and phylogenetic tree of SsMAV1 and SsDFV3 based on helicase sequence. (A) The genome organizations of SsMAV1. Open reading frames (ORFs) are shown as boxes. (B) A maximum likelihood phylogenetic tree was constructed based on amino acid alignments of helicase. The viruses in different families belong to alpha-virus subgroup were selected. Bootstrap values (%) obtained with 1000 replicates are indicated on the branches, and branch lengths correspond to genetic distance; the scale bar at the lower left corresponds to genetic distance. The viruses SsMAV1 and SsDFV3 are highlighted with red font.

The ML phylogenetic trees based on two conserved domains of Mtr and RdRp revealed that SsDFV3 is clustered with members within *Deltaflexiviridae*, suggesting that SsDFV3 is a new member within *Deltaflexiviridae* (Figs 2D and S4). Multiple alignments and phylogenetic analysis of two Hel sequences derived from SsDFV3 indicated that these two helicase domains belong to superfamily 1 (SF1) (S5A Fig). But the identity between the two helicases of SsDFV3 is 22%, and phylogenetic analysis indicated the two helicases are clustered with different branches (Fig 3B), suggesting that SsDFV3 may be obtained the second helicase via horizontal gene transfer event.

The stability and distribution of SsDFV3 in *S*. *sclerotiorum* were assayed. Three RNA segments (SsDFV3S1-S3) were recently detected in a previously characterized *S*. *sclerotiorum* strain SCH941 and its single protoplast isolate (S2B Fig). Moreover, seven of hundred *S*. *sclerotiorum* isolates isolated from a single crop field were also infected with this virus with the three fragments (S2C and S2D Fig) [5], which suggested that SsDFV3 could be widespread in *S*. *sclerotiorum*. In addition, when strain SX276 was dual-cultured with strain SCH941A1R (the virulent isolate), the three RNA segments of SsDFV3 could be horizontally co-transmitted to strain SCH941A1R (S6 Fig). The above results supported that three RNA segments should be associated with each other and form a tripartite SsDFV3 genome.

### An uncharacterized mycovirus has evolved two helicases via a gene duplication event

SsMAV1 was identified with a (+)ssRNA genome size of 7,531 nt, excluding the poly-A tail. A putative large ORF was found, and it putatively encoded a 2,435 aa polyprotein (Fig 3A). This polyprotein contains three conserved domains, including Mtr, Hel, and RdRp (Fig 3A). The RdRp of SsMAV1 shared 33% identity to that of Rhizoctonia solani alphavirus-like virus 1, whereas the Mtr of SsMAV1 showed 30% identity to that of Sclerotinia sclerotiorum RNA virus L. To define the relatedness between SsMAV1 and other viruses associated with alphavirus, the conserved domains RdRp, Mtr, and Hel were aligned, and then the phylogenetic trees were constructed (Figs 3, S7, and S8). Although SsMAV1 is related to the alphaviruses, SsMAV1 and its related mycoviruses are markedly distant from the members of all the approved families, and therefore form an independent and well-supported phylogenetic group at the evolutionary level. We, therefore, propose to establish a new family, Mycoalphaviridae, and genus, Scleroalphavirus, to accommodate those alphavirus-like viruses infecting fungi.

The previous discovery of scleroalphaviruses encoded a single polyprotein, including three conserved domains of Mtr, Hel, and RdRp. Interestingly, the polyprotein of SsMAV1 has two viral RNA helicase domains (helicase 1 and helicase 2) belonging to helicase superfamily 1 and clustered into an evolutionary branch (Figs 3B and S5B), suggesting that the duplication event of helicase had occurred in course of the SsMAV1 evolutionary history.

### The first recorded rhabdovirus infecting fungi represents an independently viral evolutionary branch

Rhabdoviruses have been reported to infect plants, invertebrates, and vertebrates including humans. Some of the rhabdoviruses are associated with harmful disease outbreaks in the fields of agriculture, veterinary and human health [36]. However, their existence in fungi has rarely been recorded [37,38]. Here, a rhabdovirus that infected fungus was identified and its complete genome was characterized.

#### Genome organization of a fungal rhabdovirus Sclerotinia sclerotiorum rhabdovirus 1

**Fig 4:**
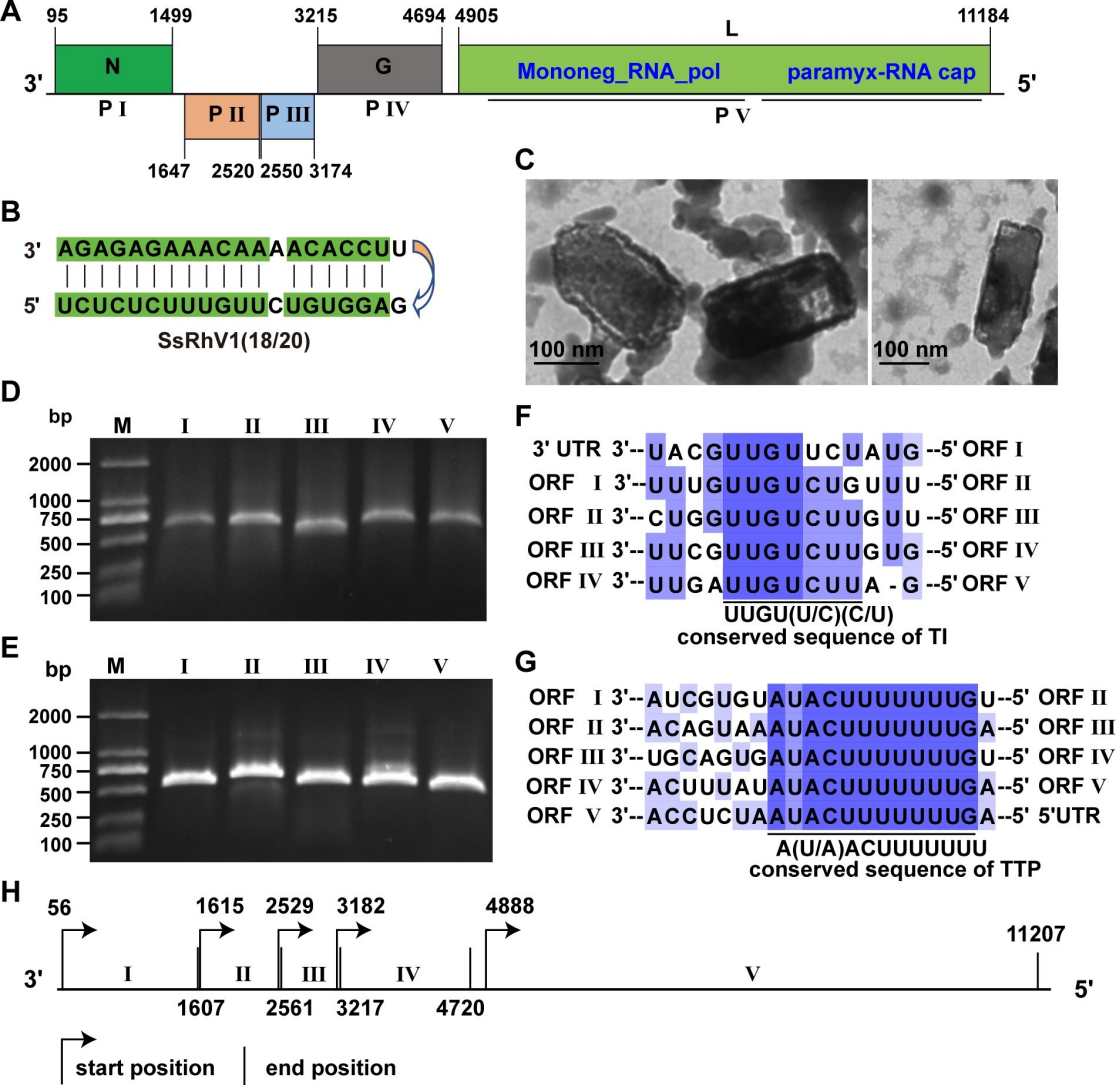
Genomic organization of SsRhV1. (A) Genome size and organization of SsRhV1. ORFs I, IV and ORF V are in the same frame, whereas ORF II and ORF III are in another frame. Boxes on the genome indicate the position and size of each ORF, which are labeled with Roman numerals. (B) The 3′ leader and 5′ trailer sequences of the SsRhV1 show terminal complementarity. (C) Morphology and structure of SsRhV1 particles. Particles were purified from mycelia of strain SX276 and negatively stained with 2% PTA [(wt/vol), pH 7.4]. (D) Agarose gel electrophoresis of the 5′ RACE of ORFs I-V on 1% agarose gel. (E) Agarose gel electrophoresis of the 3′ RACE of ORFs I-V on 1% agarose gel. (F) Comparison of transcription initiation (TI) signal between the ORFs in SsRhV1. The alignment of the transcription initiation sequences is shown in a 3′ to 5′ orientation. Conserved sequences are highlighted in purple. Different shades indicate the levels of conserved sequence, and the darkest color shade shows the most conserved sequence. (G) Comparison of transcription termination/polyadenylation (TTP) signal between the ORFs in SsRhV1. The alignment of the transcription termination/polyadenylation sequence is shown in a 3′ to 5′ orientation. (G) The possible transcript map of SsRhV1 based on the 5′ and 3′ RACE.

SsRhV1 related to the viruses within the family *Rhabdoviridae* was detected in strain SX276. Small RNA data analysis and conventional experiments including single-protoplast isolation, successive subculturing, and dual-culture indicated that SsRhV1 could infect stably and transmit horizontally in *S*. *sclerotiorum* (S6 and S9 Figs). The entire genome of the SsRhV1 is 11,356 nt in length, with a long 5′ trailer sequence of 173 nt and a short 3′ leader sequence of 97 nt (Fig 4A). The terminal sequences of the 3′ leader and 5′ trailer of the SsRhV1 showed terminal complementarity (Fig 4B). SsRhV1 has five major ORFs (ORFs I-V), and these non-overlapping ORFs are linearly arranged in the antigenomic strand and are separated by intergenic regions (Fig 4A). Electron microscopy of the purified preparations of SsRhV1 showed bullet-shaped virions, sized approximately 100–170×248–365 nm (n = 23, Fig 4C).

Five ORFs in SsRhV1 encodes five putative proteins. ORF I encodes a putative 467 aa-protein with 52.98 kDa. Blastp results revealed that the ORF I-encoded protein shares a 29% identity to the nucleocapsid protein (N) of the Beatrice Hill virus in the genus *Tibrovirus* (S3 Table). ORF IV encodes a 493 aa-protein with 56.68 kDa and has 32% identity to the glycoprotein (G) of Mount Elgon bat virus within the genus *Ledantevirus*. The ORF V encodes a larger 2093-aa protein (L protein). This protein has a 35% identity to the L protein of the Piry virus in the genus *Vesiculovirus* (S3 Table). The L protein of SsRhV1 contains two conserved domains (Fig 4A): one is Mononeg_RNA_pol (pfam00946), a typical conserved domain of members in the order *Mononegavirales* [39], and another, paramyx_RNAcap (TIGR04198), which is vital for the formation of the mRNA cap structures [40]. Thus, ORFs I, IV, and V of SsRhV1 encode the N protein, G protein, and L protein, respectively. ORF II and ORF III encode two small hypothetical proteins of 32.7 kDa and 24.3 kDa, respectively. ORF II-encoded protein is similar to a protein in *Plasmodium ovale curtisi* with low credibility (E value = 0.94, 26% identity), while the protein encoded by ORF III does not have any conserved information, suggesting that the functions of ORF II and ORF III-encoded proteins remain unknown (S3 Table).

All five genes of SsRhV1 could be transcribed independently (Fig 4D and 4E), and their transcript maps were determined via the RACE method (Fig 4H). SsRhV1 has relatively conserved transcription initiation (TI) and transcription termination/polyadenylation (TTP) sequences in the gene junction region, which is a hallmark of the members within the family *Rhabdoviridae* (Fig 4F and 4G). Notably, no conserved non-transcribed intergenic dinucleotide was found in the TI signal and TTP signal in SsRhV1. Additionally, the TI signal of the ORF II and ORF III is located upstream to the TTP of ORF III and ORF IV, respectively (Fig 4H). The overlapping of the transcription termination/polyadenylation signals also had been found in bovine ephemeral fever virus and sigmaviruses, which were significantly different from the canonical transcription map of the reported members in the family *Rhabdoviridae* [19,41,42].

#### SsRhV1 is phylogenetically related to members within the family *Rhabdoviridae*

**Fig 5:**
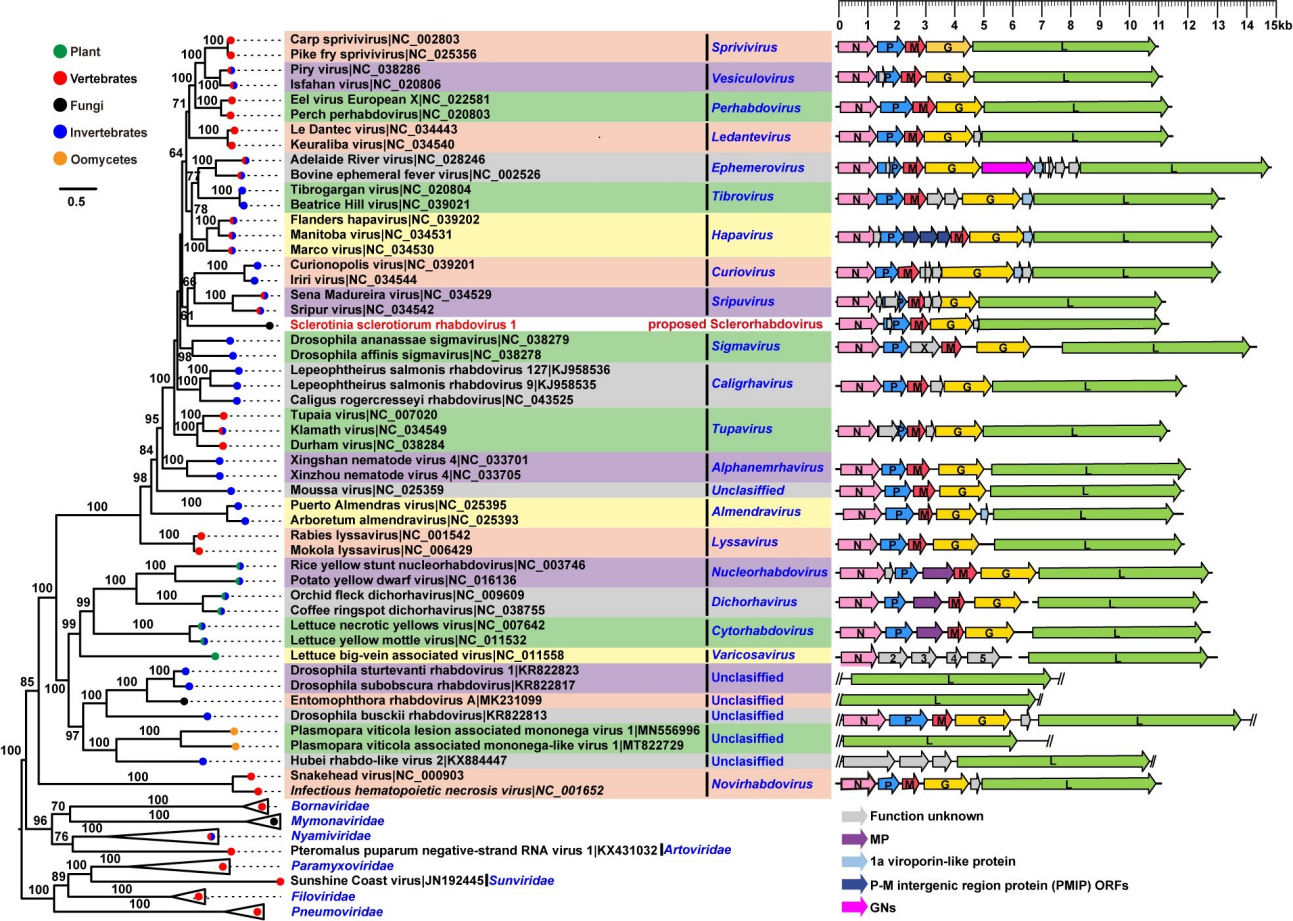
Schematic representation of genome organization and maximum likelihood tree of SsRhV1. (A) Phylogenetic analysis of SsRhV1. A maximum likelihood phylogenetic tree was constructed based on amino acid alignments of L protein. The virus SsRhV1 is represented by a red font. (B) Comparison of the organizations SsRhV1 and other viruses in the *Rhabdoviridae*. Each arrow indicates the position of a long open reading frame (ORF) (ORF >180 nt), ORFs are shown as boxes, the putative proteins are represented in the different color. Every virus has five common structural protein genes, pink indicates N protein, blue indicates P protein, orange-red indicates M protein, yellow indicates G protein, and green indicates L protein. Viroporin-like proteins are shaded in light blue. Move proteins are shaded in purple. ORFs for which no orthologous or structurally similar proteins could be identified are shaded in light grey or white.

To guide the evolutionary relationships of SsRhV1 with other viruses belonging to the family *Rhabdoviridae* with more precision, the conserved polymerase domains were aligned (S10 Fig). The phylogenetic tree suggested that the selected rhabdoviruses were distributed in 26 distinct monophyletic groups (Fig 5A). The SsRhV1 independently formed a separate monophyletic branch, and clustered with genera *Sripuvirus* and *Curiovirus* (Fig 5A). Moreover, the phylogenetic trees were constructed based on multiple alignments of N and G proteins (S11 and S12 Figs). The N protein of SsRhV1 was clustered with members within genera *Alphanemrhavirus*, *Lyssavirus*, *Almendravirus* and one unclassified virus Moussa virus, but formed an independently monophyletic branch (S11 Fig). The G protein of SsRhV1 was clustered with members in genus *Almendravirus*, but formed an independently monophyletic clade (S12 Fig). These results substantiated that SsRhV1 does not belong to any known genera but forms a single new rhabdovirus branch in the family *Rhabdoviridae*.

Except for the five canonical protein (3′-N-P-M-G-L-5′) genes in rhabdoviruses, additional smaller ORFs (≥ 180 nt) were recently discovered [19,43]. To understand the difference of additional shorter ORFs between SsRhV1 and other rhabdoviruses in size and location, the genomic organization of typical species in 20 known genera was compared (Fig 5B). The position of the additional ORFs in SsRhV1 has much in common with that of the members from the genera *Sripuvirus* and *Curiovirus* (Fig 5B). SsRhV1 has a smaller ORF in ORF II with a length of 189 nt, and the Chaco virus (NC_034550) within the genus *Sripuvirus* contains a smaller ORF in ORFII (P protein) with a length of 551 nt. Curionopolis virus (NC_039201) within the *Curiovirus* genus has an ORF with a length of 279 nt between the G and L proteins, which is a viroporin-like protein found in ephemeroviruses, tibroviruses, hapaviruses, almendraviruses, and curioviruses. Small ORF (G1) of 174 nt between the G and L proteins was found in SsRhV1. Similar to the viroporin-like protein in other rhabdoviruses, G1 protein has a transmembrane region and a highly basic C-terminal, which indicated that G1 protein might have the same function as the known viroporin-like protein (S13A Fig). The results of the 5′ RACE and 3′ RACE indicated that G1 could successfully transcribe as mRNA (S13B Fig).

### A novel endornavirus SsEV3 is a main hypovirulent factor in *S*. *sclerotiorum*

SsEV3 has a (+)ssRNA genome with 12,661 nt in length, and is composed of a single ORF. SsEV3 encodes a 4,184 aa polyprotein containing conserved domains of Mtr, Hel, phytoreo_S7 (S7), and RdRp (S14A Fig). The RdRp of SsEV3 shared 51% identity to that of Sclerotinia sclerotiorum endornavirus 1 (SsEV1, NC_023893). SsEV3 was positioned in the fungal endornavirus group (S14B Fig).

**Fig 6:**
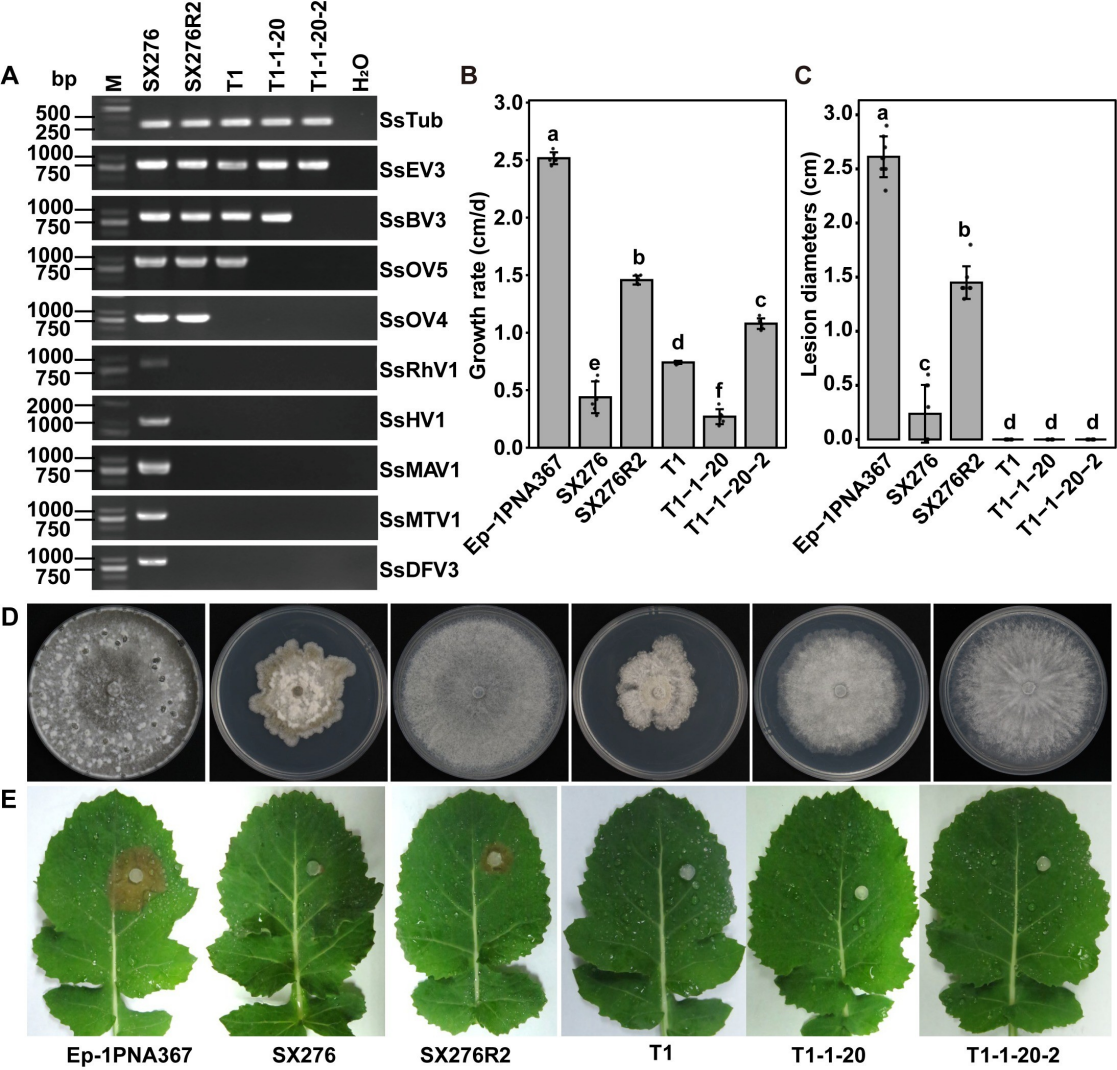
SsEV3 was responsible for hypovirulence on *S*. *sclerotiorum*. (A) Acquisition of regeneration strains through protoplasts of virus-cured method with ribavirin and cycloheximide. Strain SX276 is the original strain infected with nine viruses. Strain T1-1-20-2 was detected with a single virus SsEV3. (B) and (C) are the mathematical statistics of growth rate and diameter of leave lesion (48 h). Error bars indicate the SD from six sample means. The different letters on the top of each column showed a significantly difference at the P < 0.05 level of confidence according to the t-test. (D) The colony morphology of the strains and Ep-1PNA367(7 dpi). (E)The pathogenicity of individual isolates was assayed.

To verify the relationship between mycoviruses and hypovirulence in *S*. *sclerotiorum*, a serial strategy was applied to eliminate one or more mycoviruses from the strain SX276 (S15 Fig). The strain SX276R2 was regenerated from protoplasts of strain SX276, and was confirmed to be co-infected by four mycoviruses (SsEV3, SsBV3, SsOV4, and SsOV5) (Fig 6A). To further eliminate mycoviruses, the mycelia of the strains, SX276R2 and Ep-1PNA367R, were mixed and cultured in the same PDA plate, and then transferred for culturing on a fresh PDA plate supplemented with hygromycin B. The two new isolates, SX276R2/A367R1 and SX276R2/A367R2, were successfully isolated and confirmed to carry three mycoviruses, SsEV3, SsBV3, and SsOV5. To avoid the T-DNA insertion mutation effect, three mycoviruses SsEV3, SsBV3, and SsOV5 were successfully induced into strain Ep-1PNA367 via dual-culturing with strains SX276R2/A367R1 and SX276R2/A367R2, respectively. The newly infected strains were named Ep-1PNA367T1 and Ep-1PNA367T2, and three mycovirus content was confirmed by mycovirus-specific RT-PCR (Fig 6A). Ep-1PNA367T1 was further treated with a combination of two methods of chemical antiviral agents (cycloheximide and ribavirin) and single protoplast isolation. After two successive cycles of treatment, the strain T1-1-20-2 lacking SsBV3 and SsOV5 was successfully obtained to be exclusively infected by SsEV3 (Fig 6A). Compared to the normal strain Ep-1PNA367, all five *S*. *sclerotiorum* isolates infected with the different combinations of mycoviruses showed hypovirulent phenotypes (Fig 6D). Four isolates SX276R2 (1.46 cm/d), T1 (0.27 cm/d), T1-1-20 (0.74 cm/d), and T1-1-20-2 (1.08 cm/d), showed significantly attenuated growth, compared to the growth rate of Ep-1PNA367 (2.48 cm/d) (Fig 6B). Notably, those SsEV3-infected isolates showed lower virulence on the detached leaves (Fig 6C and 6E), and the strain SX276R2 (infected with SsEV3, SsOV5, SsBV3 and SsOV4) showed a moderate virulence. We deduced the coinfection with SsOV4 could weaken in some degree the hypovirulence induced by SsEV3.

To further determine whether SsEV3 was responsible for the hypovirulence to *S*. *sclerotiorum*, the strain T1-1-20-2 was dual-cultured with SCH941A1R on the same PDA plate for ten days (S16A Fig). Three new strains (SCH941A1RV1, SCH941A1RV2, and SCH941A1RV3) derived from SCH941A1R were screened on PDA containing hygromycin B, and their mycovirus content was confirmed by RT-PCR (S16B Fig). SsEV3 was successfully transferred into SCH941A1R via hyphal anastomosis. Significant growth and virulence reduction were observed in the new SsEV3-infected strains compared to the strain SCH941A1R, and they showed abnormal colony morphology that was similar to strain T1-1-20-2 (S16 Fig). We thereby confirmed the significance of SsEV3 as a factor for hypovirulent phenotypes in *S*. *sclerotiorum*.

## Discussion

The continuous reporting of novel mycoviruses implies the possible presence of numerous yet unrecognized mycoviruses. Further identification and clarification of the genomic characteristics of new mycoviruses will improve our understanding of their evolutionary basis and relationships with other known viruses. In the present study, nine RNA mycoviruses co-infecting a single hypovirulent strain SX276 of *S*. *sclerotiorum* were identified. The newly identified mycoviruses not only included four previously characterized mycoviruses, but also two distant relatives of known mycoviruses. As well, we provide the first report of a segmented deltaflexivirus containing two distant helicases and a rhabdovirus infecting fungi. Nine mycoviruses belonging to eight potential virus families show that *S*. *sclerotiorum* strain SX276 harbors a complex and diverse mycovirus community.

The discovery of SsDFV3 demonstrates a new example for insights into the evolution of viral genome segmentation. The genomes of RNA viruses are divided into segmented or unsegmented genomes [44]. The genome segmentation from ancestral unsegmented RNA genomes probably occurred independently during viral evolution. The actual evolutionary benefits of genome segmentation are still poorly understood; however, these events might play an important role in the evolutionary history of these viruses. Segmented viruses are generally considered to be evolved from unsegmented viruses, since segmented viruses have several survival advantages in evolution [6,44–47]. Researchers have been trying to uncover evidence to support this conclusion. The members within *Chuviridae* display genomic features of both segmented and unsegmented RNA viruses, which provides a new perspective on the evolutionary origins of segmented and unsegmented viruses [47]. The Jingmen tick virus (Jingmenvirus) has four segmented genomes, and its two segments are derived from unsegmented flaviviruses, which represents an evolutionary change from unsegmented to segmented viruses. Subsequently, the jingmenvirus was confirmed to be widely distributed in the world [48]. However, jingmenviruses are phylogenetically clustered in a separate group that is distinctive from the genera of *Flavivirus* [49]. Recently, the novel narna-like viruses and virga-like mycovirus with segmented genomes have been found in *Aspergillus species*, *S*. *sclerotiorum*, and endomycorrhizal fungi, suggesting that mycovirus genome segmentation are more complex [5–7]. From our present study, SsDFV3 has a tripartite genome, and was phylogenetically positioned into previously characterized unsegmented deltaflexiviruses group based on conserved RdRp domain. The proteins encoded by the S2 segment of SsDFV3 were similar to proteins encoded by the unsegmented FgDFV1 and SsDFV1. Thus, we speculate that the SsDFV3S2 may have originated from an unsegmented deltaflexivirus via fragmentation, and that SsDFV3 represents a critical evolutionary position that organically links segmented and unsegmented viruses, and directly provides evolutionary evidence for virus genome segmentation. S3 RNA segment of SsDFV3 shared no significant sequence or structural homology with known deltaflexiviruses, but to relate members within the family *Benyviridae* and the proposed family Mycoalphaviridae. The analysis results of 3’ terminal structures of the SsDFV3 genome indicated that the SsDFV3 S2 and S1 segment have a poly(A) tail structure, while the S3 segment lacks the poly(A) tail. It is supposed that segments from the same virus should have the same structure to follow the same strategy for translation. Our finding could support that the S3 segment belongs in its origin to a different virus, and that was acquired by a bi-segmented SsDFV3 virus. We speculated that SsDFV3 has co-infected or been co-infecting a fungus host with mycoalphaviruses including SsMAV1, and the genome recombination or reassortment has occurred between the two viruses and then resulting in a unique genome organization for SsDFV3. But, the functions of the two small segments are still unknown. For RNA viruses, reassortment and recombination were perceived as the primary means of promoting genetic exchange, purging deleterious mutations, and creating new advantageous combinations [50]. However, we previously reported that SsDFV2 has a monopartite RNA genome containing a single large ORF for replicase [51]. Thus, it is difficult to exclude a possibility that the ancestral deltaflexivirus had an SsDFV2-type genome, and it acquired a small gene or a gene set during evolution.

SsMAV1 and SsDFV3 harbor two helicase genes. Two helicases of SsDFV3 are encoded by two different RNA segments and clustered into two phylogenetical groups, while two helicases of SsMAV1 are located in a single RNA segment and have a close relationship with each other. Helicases from viral genomes are usually divided into three superfamilies (SF1 to 3) according to the characteristics of their conserved motif [52]. All helicases from SsDFV3 and SsMAV1 belonged to SF1. The phenomenon of two helicases in the same virus has been reported in plant viruses and mycoviruses [53,54]. In plant viruses, the second helicase evolved to new biological functions, including viral silencing suppressor and/or inter-cell movement [53,55]. In mycoviruses, rarely endornaviruses have infrequently contained two helicases, but differing from SsDFV3 and SsMAV1, they respectively belong to SF1 and SF2 [56]. A fusarivirus in *Rhizoctonia solani* was recently reported to contain two SF2 helicases located in two ORFs [57]. The two helicases of SsDFV3 were located on the different evolutionary branches, while both two helicases derived from SsMAV1 were close to that of mycoalphaviruses. The possible mechanisms of the second helicase gene evolution in plant viruses include two proposed scenarios: one is the combination of horizontal transfer; another is gene duplication [55]. SsMAV1 was considered to possess two helicases through duplication of a replicative RNA helicase, while SsDFV3 acquired an additional helicase via horizontal gene transfer. Similar results with SsDFV3 were recently detected in a tripartite virga-like mycovirus that contains a dual methyltransferase domain encoded by two RNA segments, and phylogenetically positioned into two evolutionary clades [6].

Rhabdoviruses have been identified in various species, but no cases of these viruses infecting fungi have been recorded. Some rhabdovirus-related sequence has been reported in the virome of the fungal-like oomycete *Plasmopara viticola* and public fungal transcriptomes [37,38], but SsRhV1 should be the first reported rhabdovirus that infected a true fungus. Although a similar genome structure was described between SsRhV1 and the majority of known rhabdoviruses, SsRhV1 also has shown some unique features in the genome and evolutionary process. Firstly, the transcription strategy of SsRhV1 is different from the known rhabdoviruses. Usually, the mRNAs of rhabdoviruses have a 5′-cap structure and 3′-polyadenylated tails, and an internal cis-acting stop and restart signals between them, but SsRhV1 may not follow this rule, and no conserved non-transcribed intergenic dinucleotide between the conserved TI and TTP. The sequence of the TI signal in SsRhV1 is 3′-UUGU(C/U)-5′, while the sequence is 3′ -UUGUC-5′ in vesicular stomatitis viruses. The sequence of TTP in SsRhV1 is 3′-A(U/A)ACUUUUUUU-5′, whereas the sequence in VSV is 3′-AUACUUUUUUU-5′ [19]. Secondly, based on the phylogenetic tree constructed with three proteins (L, N, and G proteins), SsRhV1 formed a distinct monophyletic branch that is significantly distant from the known 20 genera within *Rhabdoviridae*. Thirdly, both the ORF II and III of SsRhV1 encode hypothetical proteins without any conserved domains, whereas the corresponding two ORFs encode for the phosphoprotein and matrix protein in the reported rhabdoviruses. In addition, except for the varicosavirus that is transmitted by the chytrid fungus *Olpidium virulentus* [58,59], hosts of other reported rhabdoviruses are animals or plants. Therefore, we proposed to establish a new genus, Sclerorhabdovirus, to accommodate SsRhV1 and its related but unidentified rhabdovirus. The discovery of SsRhV1 has made the map of the classification of *Rhabdoviridae* more complete, since rhabdoviruses, so far, have been confirmed to infect members from three kingdoms of animals, plants, and fungi. Identification of SsRhV1 enriched the diversity of rhabdoviruses and supplied new evolutionary clues to their origins. Unfortunately, our study failed to obtain strains infected by SsRhV1 alone via multiple methods of elimination and transfection. Thus, the biological effects of SsRhV1 on *S*. *sclerotiorum* remain to be explored in future research.

Endornaviruses were reported to infect plants, fungi and possibly insects, and have no significant impact on fungi [60], except for the HmEV1 and SmEV1 [34,35]. HmEV1 was identified from a hypovirulent strain of *Helicobasidium mompa*, but whether HmEV1 is associated with hypovirulence remains to be explored. SmEV1 was associated with hypovirulence in *Sclerotinia minor*. SsEV3 conferred hypovirulence to *S*. *sclerotiorum*. Of note, the debilitated traits (abnormal colony morphology and decreased growth rate) of strains infected by SsEV3 alone were milder than those observed for strain SX276. It may be the result of the different genetic backgrounds of these strains or the result of the co-infection of multiple-mycovirus in strain SX276 and further studies are needed to determine whether infection by multiple viruses contributes to greater hypovirulence.

## Material and methods

### Fungal strains and culture conditions

*S*. *sclerotiorum* strain SX276 was isolated and purified from a sclerotium collected from diseased rapeseed in Shanxi province, P.R. China. The two virus-free strains Ep-1PNA367 and SCH941A1 were obtained from single ascospore isolation from two hypovirulent strains Ep-1PN and SCH941, respectively [61,62]. The strains Ep-1PNA367R and SCH941A1R were labeled with the *hygromycin* B resistance gene and served as the recipient strain for horizontal transmission assays. The strains Ep-1PNA367, Ep-1PNA367R, SCH941A1R, and SCH941A1 have normal biological features, including strong virulence on their hosts. All *S*. *sclerotiorum* strains were cultured on potato dextrose agar (PDA) at 20°C, and maintained on PDA slants at 4°C.

### Total RNA extraction, dsRNA extraction, and sequencing

Strain SX276 was cultured on a cellophane membrane overlaying the PDA for 5 days. Mycelium (approximately 1 g) was collected and then ground to a powder in liquid nitrogen. The dsRNA was extracted as previously described [28], and followed by purification to remove DNA and ssRNA by digestion with S1 nuclease and DNase I (TaKaRa, Dalian, China) [28]. The dsRNA samples were used for mycovirus detection and mycoviral terminal sequence determination.

Total RNA was prepared using a Trizol RNA extraction kit (TaKaRa, Dalian, China) according to the manufacturer’s instructions and treated with DNase I. The total RNA was used for metatranscriptomic sequencing using the rRNA depletion method. Sequencing was performed on the Illumina MiSeq 2000/2500 (Shanghai Biotechnology Corporation, Shanghai, China), and the following analysis was consistent with the previous description [63]. Deep sequencing of small RNA (sRNA) was performed on the Illumina HiSeq 50 (Novogene Company, Beijing, China), the terminal of sRNA was ligated with the adapter, then reverse-transcribed to synthesize cDNA. After PCR amplification, polyacrylamide gel electrophoresis (PAGE) was used to separate the target DNA fragments. The purified DNA fragments were used for sequencing. The following analysis process was consistent with the previous description [64].

### Mycoviruses confirmation and their full-length determination

Raw data were trimmed by using Trimmomatic program (version 0.36) [65] with default parameter settings, and the clean reads were used for data analysis. HISAT2 was used to map reads against the genome of *S*. *sclerotiorum* (http://fungi.ensembl.org/Sclerotinia_sclerotiorum_1980_uf_70_gca_001857865/Info/Index) [66]. The reads that did not match with the genome were extracted for further analysis. The reads were then assembled *de novo* using the SPAdes with default parameter settings [67]. The assembled contigs were compared (using BLASTx) against the GenBank nr database with an e-value of 1 × 10^−5^ to maintain high sensitivity and a low false-positive rate.

To verify the presence of putative mycoviruses in the strain SX276, cDNAs were synthesized by using Moloney murine leukemia virus transcriptase (M-MLV, Takara Dalian, China) with hexdeoxyribonucleotide mixture random primers (Takara Dalian, China). To ensure the accuracy of the mycovirus sequences from the metatranscriptomic data, specific primers (S4 Table) for individual mycoviruses were designed based on the assembled contigs. The length of each PCR product for individual mycoviruses was approximately 700–2,000 bp, containing an overlap between adjacent segments. The expected PCR products were purified and cloned into the pMD18-T vector to be re-sequenced. A single primer amplification method with minor modifications [68] was used to obtain the terminal sequence of all mycoviruses infecting strain SX276. The dsRNA (200–500 ng) or total RNA (2–5 μg) were mixed with 30 pmol primer PC3-T7 loop (5′-p-GGATCCCGGGAATTCGGTAATACGACTCACTATATTTTTATAGTGAGTCGTATTA-OH-3′). The first cDNA synthesis was conducted using the M-MLV transcriptase according to the manufacturer’s instructions. PCR amplification was performed using the PC2 primer (5′-p-CCGAATTCCCGGGATCC-3′) complementary to the primer PC3-T7 loop and the sequence-specific primer corresponding to the 5′-and 3′-terminal sequences of individual mycoviruses, respectively. The expected PCR products were purified and sent to the company for sequencing.

The 5′ and 3′ rapid amplification of cDNA ends (RACE) techniques were used to determine the transcription strategy of a negative-sense RNA virus, SsRhV1. Firstly, the total RNA samples of SX276 were extracted from mycelia growing on PDA for 4–6 days, and the mRNA samples were isolated with a Magnosphere UltraPure mRNA Purification Kit (Takara Dalian, China). Then, these mRNA samples were used to obtain the sequence of 5′ and 3′ RACE experiments using the methods described previously [69]. The primers used for RACE analyses are listed in S5 Table.

### The mycoviral genome analysis and phylogenetic analysis

The basic features of the full-length genomes of mycoviruses and potential ORFs were analyzed using the DNAMAN 8 software. The conserved motifs were predicted using a motif search website i.e., http://www.genome.jp/tools/motif/. Phylogenetic analysis was conducted based on a maximum-likelihood (ML) method as described previously with minor modifications [43]. The amino acid sequences of viruses were aligned using the MAFFT 7.0 software, as described previously [70]. The viruses used in the alignment analysis were listed in S6 Table. Removal of the poorly aligned regions was conducted using trimAl [71]. Subsequently, PhyML 3.0 was used to construct an ML phylogenetic tree with automatic model selection (http://www.atgc-montpellier.fr/phyml/) [72]. Branch supports were estimated based on 1000 bootstrap repetitions. The phylogenetic trees were visualized using Figtree 1.4.4 software.

### Phenotypic measurements of *S*. *sclerotiorum* strains

The biological characteristics of the strains in this study including growth rate, virulence, and morphology observation of the hyphal tips were assessed as previously described [17]. There were more than three replicates for each treatment, and all the biological characterization experiments were conducted at least three times. The experimental data were analyzed and visualized by using RStudio. Every experimental treatment contained at least six samples and the significant difference analysis was performed at the level of P<0.05 by using a *t*-test.

### Virus particles purification, transmission electron microscopy observation, protoplast preparation, and transfection

To extract viral particles, strain SX276 was grown on cellophane membranes overlying PDA plate for five days, and the mycelia were harvested. Then virus particles were purified and visualized using transmission electron microscopy, as described previously [17]. Strain SCH941A1R served as the recipient strain. The protoplast preparation was performed as previously reported [73]. Then, the transfections with the purified virion, the total RNA samples, and dsRNA samples of SX276 were performed as described previously [3,74]. The transfectants were confirmed using PCR amplification with virus specific primers listed in S4 Table.

### Horizontal transmission of virus between *S*. *sclerotiorum* strains, protoplast generation

Strain SCH941A1R served as the recipient strain, mycoviruses-infected strains were co-cultured with the recipient strain on PDA plate (15 cm) for 10 days at 20°C. The new isolates were picked up from the colony margin of the virulent strain and transferred on a fresh PDA plate supplemented with hygromycin B, after three generations the RT-PCR was performed to detect the viruses. The protoplast preparation and viral elimination were performed as described previously [73,75].

### Northern blot hybridization

The probes are amplified and purified with the primers listed in S1 Table, and then labeled following the instruction of Gene Images AlkPhos Direct Labelling and Detection System (Amersham, United Kingdom). The northern blot was carried out according to the instructions of Gene Images AlkPhos Direct Labelling and Detection System and Hybond-N^+^ (Amersham, United Kingdom). The Chemiluminescent signal generation and detection with the CDP-Star Detection Reagent (Amersham, United Kingdom).

## Supporting information

S1 Fig Schematic representation of genome organization and maximum likelihood tree of SsOV4/SX276 and SsOV5. (A) Schematic organization and annotations of SsOV4/SX276 and SsOV5 genome. Open reading frames (ORFs) are shown as boxes, the conserved motifs were represented in different colors. (B) Phylogenetic analysis of SsOV4/SX276 and SsOV5. A maximum-likelihood phylogenetic tree was constructed based on amino acid alignments of RdRp. Bootstrap values (%) obtained with 1000 replicates are indicated on the branches, and branch lengths correspond to genetic distance; the scale bar at the lower left corresponds to genetic distance. The novel viruses of SsOV4/SX276 and SsOV5 are highlighted with red font.
(TIF)

S2 Fig The stability and distribution of virus SsDFV3 in *S*. *sclerotiorum*. (A) Detection of SsDFV3S1-S3 in protoplast regeneration strains of SX276. (B) Detection of SsDFV3S1-S3 in protoplast regeneration strains of SCH941. (C) RT-PCR was carried out to determine the group of segments S1-S3 among 100 isolates of *S*. *sclerotinia* from a single crop field collected in May 2017. C1-C20 represent twenty groups, and each group contains five strains. (D) RT-PCR was performed to determine the strains of SsDFV3S1-S3.
(TIF)

S3 Fig The alignment of SsDFV3 Hel-2. (A) The 5 most similar viruses with the Hel-2 of SsDFV3 in Protein Data Bank (PDB). (B) Sequence alignment of the Hel-2 domain from SsDFV3 and Chrysanthemum virus B.
(TIF)

S4 Fig Maximum likelihood tree of SsMTV1/SX276 and SsDFV3 based on the amino acid sequence of methyltransferase. Phylogenetic analysis of SsMTV1/SX276 and SsDFV3 constructed based on amino acid alignments of viral methyltransferase sequence. The tree was constructed by a maximum likelihood method. The novel viruses SsMTV1/SX276 and SsDFV3 are highlighted with red font.
(TIF)

S5 Fig Multiple alignments of viruses SsMTV1, SsDFV3, and SsMAV1 based on helicase sequence. (A) Multiple alignments based on the helicase amino acid sequence of SsMTV1/SX276 and SsDFV3 and other viruses in order *Tymovirales*. (B) Multiple alignments based on the helicase amino acid sequence of SsMAV1 and other viruses in alphavirus sub-group.
(TIF)

S6 Fig Stability and transmission of SsRhV1 and SsDFV3. (A) Co-culture of strain SX276 and SCH941A1R (15 dpi). The new isolates (indicated by blue circles) were picked up from the colony margin of the virulent strain. (B) The colony morphology of the virus recipient strains (7 dpi). (C) The detection of the SsRhV1 and SsDFV3 in the virus recipient strains. (D) RT-PCR was performed to detect the SsRhV1 in the protoplast isolations from strain SX276. (E) Detection of SsRhV1 was performed during strain SX276 subculturing. (F) Colony morphology of the protoplast isolations from strain SX276 (7 dpi). (G) Colony morphology of the strain SX276 after subculturing 1 to 5 times.
(TIF)

S7 Fig The maximum likelihood tree of SsMAV1 based on the amino acid sequence of RdRp. Phylogenetic analysis of SsMAV1. A maximum-likelihood phylogenetic tree was constructed based on amino acid alignments of RdRp. The novel virus of SsMAV1 is highlighted in red font.
(TIF)

S8 Fig Multiple alignments and maximum likelihood tree of SsMAV1 based on the amino acid sequence of methyltransferase. (A) Multiple alignments based on the methyltransferase amino acid sequence of SsMAV1 and other viruses in alphavirus sub-group. (B) Phylogenetic analysis of SsMAV1 constructed based on amino acid alignments of viral methyltransferase sequence. The tree was constructed by a maximum likelihood method. SsMAV1 is highlighted in red font.
(TIF)

S9 Fig The information of virus-derived small interfering RNAs (siRNAs) derived from SsRhV1. (A) Size distribution of vsiRNAs derived from SsRhV1. x-axis represents the size of vsiRNAs, and the y-axis shows numbers; red represents vsiRNAs derived from sense, and the cyan-blue represents vsiRNAs derived from anti-sense. (B) Distribution of the vsiRNAs mapped on the corresponding genome of SsRhV1. The x-axis shows schematic genomic organization of SsRhV1. The y-axis indicates the numbers of vsiRNAs matching the sense strand (red, above the x-axis) and antisense strand (cyan-blue, below the x-axis). (C) The relative frequency of 5’-terminal nucleotide of vsiRNAs. The x-axis represents the vsiRNAs size (nt), and the y-axis shows the percentages of 5’-terminal nucleotides consisting of G, C, A, and U in the 18- to 30-nt vsiRNAs class.
(TIF)

S10 Fig Multiple alignments of the SsRhV1. Multiple alignments based on the RdRp amino acid sequence of SsRhV1 and other viruses in family *Rhabdoviridae*.
(TIF)

S11 Fig Phylogenetic analysis of SsRhV1 constructed based on amino acid alignments of N protein. The tree was constructed by a maximum likelihood method. The virus SsRhV1 is represented by the red font.
(TIF)

S12 Fig Phylogenetic analysis of SsRhV1 constructed based on amino acid alignments of G protein. The tree was constructed by a maximum likelihood method. The virus SsRhV1 is represented by the red font.
(TIF)

S13 Fig Transmembrane domain of the G1 protein and the results of gene RACE. (A) The transmembrane domain prediction of G1 protein. (B) Agarose gel electrophoresis of the 3′ and 5′ RACE of G1 ORF on 1% agarose gel.
(TIF)

S14 Fig Genome organization and maximum-likelihood tree of SsEV3. (A) The organizations of SsEV3. Open reading frames (ORFs) are shown as boxes. (B) Phylogenetic analysis of SsEV3. A maximum-likelihood phylogenetic tree was constructed based on amino acid alignments of RdRp. SsEV3 is highlighted in red font.
(TIF)

S15 Fig Experimental procedure about co-culture of strains. (A) The procedure to the strain T1-1-20-2 only infected with SsEV3 through protoplast regeneration and dual-culture. (B) Dual-culture of strains T1-1-20-2 and SCH941A1R.
(TIF)

S16 Fig The characterization of strains infected with SsEV3. (A) Dual-culture of strains T1-1-20-2 and SCH941A1R (10 dpi), dual-culture of two SCH941A1R strains were as the control. (B) The detection of the viruses in the virus recipient strains. (C) The colony morphology of the individual strains (5 dpi). (D)The determination of pathogenicity of the individual strains. (E) and (F) the growth rate and diameter of leave lesion (48 hpi). Error bars indicate the SD from six sample means. The different letters on the top of each column indicate significantly difference at the P < 0.05 level of confidence according to the *t*-test.
(TIF)

S1 Table The primer for detection of nine viruses. (XLSX)

S2 Table The ORFs information of SsDFV3. (XLSX)

S3 Table The ORFs information of SsRhV1. (XLSX)

S4 Table The primer for the contigs. (XLSX)

S5 Table The primer for the RACE of SsRhV1. (XLSX)

S6 Table The list of viruses used in alignment analysis. (XLSX)

## References

[1] Ghabrial SA, Caston JR, Jiang D, Nibert ML, Suzuki N. 50-plus years of fungal viruses. Virology. 2015;479–480:356–368. doi: 10.1016/j.virol.2015.02.034 . 25771805

[2] Sato Y, Shamsi W, Jamal A, Bhatti MF, Kondo H, Suzuki N. Hadaka Virus 1: a capsidless eleven-segmented positive-sense single-stranded RNA virus from a phytopathogenic fungus, *Fusarium oxysporum*. mBio. 2020;11:e00450–00420. doi: 10.1128/mBio.00450-20 . 32457242 PMC7251205

[3] Kanhayuwa L, Kotta-Loizou I, Ozkan S, Gunning AP, Coutts RHA. A novel mycovirus from *Aspergillus fumigatus* contains four unique dsRNAs as its genome and is infectious as dsRNA. Proc Natl Acad Sci USA. 2015;112:9100–9105. doi: 10.1073/pnas.1419225112 . 26139522 PMC4517262

[4] Li P, Wang S, Zhang L, Qiu D, Zhou X, Guo L. A tripartite ssDNA mycovirus from a plant pathogenic fungus is infectious as cloned DNA and purified virions. Sci Adv. 2020;6:eaay9634. doi: 10.1126/sciadv.aay9634. 32284975 PMC7138691

[5] Jia J, Fu Y, Jiang D, Mu F, Cheng J, Lin Y, et al. Interannual dynamics, diversity and evolution of the virome in *Sclerotinia sclerotiorum* from a single crop field. Virus Evol. 2021;7:veab032. doi: 10.1093/ve/veab032. 33927888 PMC8058396

[6] Chiba Y, Oiki S, Yaguchi T, Urayama SI, Hagiwara D. Discovery of divided RdRp sequences and a hitherto unknown genomic complexity in fungal viruses. Virus Evol. 2021;7:veaa101. doi: 10.1093/ve/veaa101. 33505709 PMC7816673

[7] Sutela S, Forgia M, Vainio EJ, Chiapello M, Daghino S, Vallino M, et al. The virome from a collection of endomycorrhizal fungi reveals new viral taxa with unprecedented genome organization. Virus Evol. 2020;6:veaa076. doi: 10.1093/ve/veaa076. 33324490 PMC7724248

[8] Ruiz-Padilla A, Rodriguez-Romero J, Gomez-Cid I, Pacifico D, Ayllon MA. Novel mycoviruses discovered in the mycovirome of a necrotrophic fungus. mBio. 2021;12. doi: 10.1128/mBio.03705-20. 33975945 PMC8262958

[9] Marshall N, Priyamvada L, Ende Z, Steel J, Lowen AC. Influenza virus reassortment occurs with high frequency in the absence of segment mismatch. PLoS Pathog. 2013;9:e1003421. doi: 10.1371/journal.ppat.1003421. 23785286 PMC3681746

[10] Vijaykrishna D, Mukerji R, Smith GJ. RNA virus reassortment: an evolutionary mechanism for host jumps and immune evasion. PLoS Pathog. 2015;11:e1004902. doi: 10.1371/journal.ppat.1004902. 26158697 PMC4497687

[11] Zhu J, Zhu H, Gao B, Zhou Q, Zhong J. Diverse, novel mycoviruses from the virome of a hypovirulent *Sclerotium rolfsii* strain. Front Plant Sci. 2018;9:1738. doi: 10.3389/fpls.2018.01738. 30542362 PMC6277794

[12] Bartholomaus A, Wibberg D, Winkler A, Puhler A, Schluter A, Varrelmann M. Deep sequencing analysis reveals the mycoviral diversity of the virome of an avirulent isolate of *Rhizoctonia solani* AG-2-2 IV. PLoS One. 2016;11:e0165965. doi: 10.1371/journal.pone.0165965. 27814394 PMC5096721

[13] Osaki H, Sasaki A, Nomiyama K, Tomioka K. Multiple virus infection in a single strain of *Fusarium poae* shown by deep sequencing. Virus Genes. 2016;52:835–847. doi: 10.1007/s11262-016-1379-x . 27550368

[14] Thapa V, Roossinck MJ. Determinants of coinfection in the mycoviruses. Front Cell Infect Microbiol. 2019;9:169. doi: 10.3389/fcimb.2019.00169. 31179246 PMC6542947

[15] Hillman BI, Annisa A, Suzuki N. Viruses of plant-interacting fungi. Adv Virus Res. 2018;100:99–116. doi: 10.1016/bs.aivir.2017.10.003 . 29551145

[16] Xie J, Jiang D. Mixed infections of mycoviruses in phytopathogenic fungus *Sclerotinia sclerotiorum*. In: Bamford D, Zuckerman M, editors. Encyclopedia of Virology. 2021. pp. 461–467.

[17] Wu S, Cheng J, Fu Y, Chen T, Jiang D, Ghabrial SA, et al. Virus-mediated suppression of host non-self recognition facilitates horizontal transmission of heterologous viruses. PLoS Pathog. 2017;13:e1006234. doi: 10.1371/journal.ppat.1006234. 28334041 PMC5363999

[18] Zhang R, Hisano S, Tani A, Kondo H, Kanematsu S, Suzuki N. A capsidless ssRNA virus hosted by an unrelated dsRNA virus. Nat Microbiol. 2016;1:15001. doi: 10.1038/nmicrobiol.2015.1. 27571749

[19] Dietzgen RG, Kondo H, Goodin MM, Kurath G, Vasilakis N. The family *Rhabdoviridae*: mono- and bipartite negative-sense RNA viruses with diverse genome organization and common evolutionary origins. Virus Res. 2017;227:158–170. doi: 10.1016/j.virusres.2016.10.010 . 27773769 PMC5124403

[20] Jackson AO, Dietzgen RG, Goodin MM, Bragg JN, Deng M. Biology of plant rhabdoviruses. Annu Rev Phytopathol. 2005;43:623. doi: 10.1146/annurev.phyto.43.011205.141136. 16078897

[21] Kondo H, Maeda T, Shirako Y, Tamada T. Orchid fleck virus is a rhabdovirus with an unusual bipartite genome. J Gen Virol. 2006;87:2413–2421. doi: 10.1099/vir.0.81811-0 . 16847138

[22] Walker PJ, Blasdell KR, Calisher CH, Dietzgen RG, Kondo H, Kurath G, et al. ICTV virus taxonomy profile: *Rhabdoviridae*. J Gen Virol. 2018;99:447–448. doi: 10.1099/jgv.0.001020 . 29465028 PMC12662152

[23] Bolton MD, Thomma BP, Nelson BD. *Sclerotinia sclerotiorum* (Lib.) de Bary: biology and molecular traits of a cosmopolitan pathogen. Mol Plant Pathol. 2006;7:1–16. doi: 10.1111/j.1364-3703.2005.00316.x . 20507424

[24] Tian B, Xie J, Fu Y, Cheng J, Li B, Chen T, et al. A cosmopolitan fungal pathogen of dicots adopts an endophytic lifestyle on cereal crops and protects them from major fungal diseases. ISME J. 2020;14:3120–3135. doi: 10.1038/s41396-020-00744-6 . 32814863 PMC7784893

[25] Jiang D, Fu Y, Li G, Ghabrial SA. Viruses of the plant pathogenic fungus *Sclerotinia sclerotiorum*. Adv Virus Res. 2013;86:215–248. doi: 10.1016/B978-0-12-394315-6.00008-8 . 23498908

[26] Xie J, Jiang D. New insights into mycoviruses and exploration for the biological control of crop fungal diseases. Annu Rev Phytopathol. 2014;52:45–68. doi: 10.1146/annurev-phyto-102313-050222 . 25001452

[27] Yu X, Li B, Fu Y, Jiang D, Ghabrial SA, Li G, et al. A geminivirus-related DNA mycovirus that confers hypovirulence to a plant pathogenic fungus. Proc Natl Acad Sci USA. 2010;107:8387–8392. doi: 10.1073/pnas.0913535107 . 20404139 PMC2889581

[28] Liu L, Xie J, Cheng J, Fu Y, Li G, Yi X, et al. Fungal negative-stranded RNA virus that is related to bornaviruses and nyaviruses. Proc Natl Acad Sci USA. 2014;111:12205–12210. doi: 10.1073/pnas.1401786111 . 25092337 PMC4143027

[29] Wang M, Wang Y, Sun X, Cheng J, Fu Y, Liu H, et al. Characterization of a novel megabirnavirus from *Sclerotinia sclerotiorum* reveals horizontal gene transfer from single-stranded RNA virus to double-stranded RNA virus. J Virol. 2015;89:8567–8579. doi: 10.1128/JVI.00243-15 . 26063429 PMC4524227

[30] Yu X, Li B, Fu Y, Xie J, Cheng J, Ghabrial SA, et al. Extracellular transmission of a DNA mycovirus and its use as a natural fungicide. Proc Natl Acad Sci USA. 2013;110:1452–1457. doi: 10.1073/pnas.1213755110 . 23297222 PMC3557086

[31] Zhang H, Xie J, Fu Y, Cheng J, Qu Z, Zhao Z, et al. A 2-kb mycovirus converts a pathogenic fungus into a beneficial endophyte for *Brassica* protection and yield enhancement. Mol Plant. 2020;13:1420–1433. doi: 10.1016/j.molp.2020.08.016 . 32998002

[32] Khalifa ME, Pearson MN. Molecular characterisation of an endornavirus infecting the phytopathogen *Sclerotinia sclerotiorum*. Virus Res. 2014;189:303–309. doi: 10.1016/j.virusres.2014.06.010 . 24979045

[33] Marzano S-YL, Nelson BD, Ajayi-Oyetunde O, Bradley CA, Hughes TJ, Hartman GL, et al. Identification of diverse mycoviruses through metatranscriptomics characterization of the viromes of five major fungal plant pathogens. J Virol. 2016;90:6846–6863. doi: 10.1128/JVI.00357-16 WOS:000382306100019. 27194764 PMC4944287

[34] Osaki H, Nakamura H, Sasaki A, Matsumoto N, Yoshida K. An endornavirus from a hypovirulent strain of the violet root rot fungus, *Helicobasidium mompa*. Virus Res. 2006;118:143–149. doi: 10.1016/j.virusres.2005.12.004 . 16417937

[35] Yang D, Wu M, Zhang J, Chen W, Li G, Yang L. Sclerotinia minor Endornavirus 1, a novel pathogenicity debilitation-associated mycovirus with a wide spectrum of horizontal transmissibility. Viruses. 2018;10:589. doi: 10.3390/v10110589. 30373273 PMC6266790

[36] Longdon B, Murray GG, Palmer WJ, Day JP, Parker DJ, Welch JJ, et al. The evolution, diversity, and host associations of rhabdoviruses. Virus Evol. 2015;1:vev014. doi: 10.1093/ve/vev014. 27774286 PMC5014481

[37] Chiapello M, Rodríguez-Romero J, Ayllón MA, Turina M. Analysis of the virome associated to grapevine downy mildew lesions reveals new mycovirus lineages. Virus Evol. 2020;6:veaa058. doi: 10.1093/ve/veaa058. 33324489 PMC7724247

[38] Jo Y, Choi H, Chu H, Cho WK. Identification of viruses from fungal transcriptomes. bioRxiv. 2020:2020.2002.2026.966903. doi: 10.1101/2020.02.26.966903

[39] Banerjee AK, Barik S, De BP. Gene expression of nonsegmented negative strand RNA viruses. Pharmacol Ther. 1991;51:47–70. doi: 10.1016/0163-7258(91)90041-j . 1771177

[40] Liang B, Li Z, Jenni S, Rahmeh AA, Morin BM, Grant T, et al. Structure of the L protein of vesicular stomatitis virus from electron cryomicroscopy. Cell. 2015;162:314–327. doi: 10.1016/j.cell.2015.06.018 . 26144317 PMC4557768

[41] Teninges D, Bras F, Dezélée S. Genome organization of the sigma rhabdovirus: six genes and a gene overlap. Virol J. 1993;193:1018–1023. doi: 10.1006/viro.1993.1219 . 8384742

[42] McWilliam SM, Kongsuwan K, Cowley JA, Byrne KA, PJ W. Genome organization and transcription strategy in the complex GNS-L intergenic region of bovine ephemeral fever rhabdovirus. J Gen Virol. 1997;78:1309–1317. doi: 10.1099/0022-1317-78-6-1309 . 9191923

[43] Walker PJ, Firth C, Widen SG, Blasdell KR, Guzman H, Wood TG, et al. Evolution of genome size and complexity in the *Rhabdoviridae*. PLoS Pathog. 2015;11:e1004664. doi: 10.1371/journal.ppat.1004664. 25679389 PMC4334499

[44] Michalakis Y, Blanc S. The curious strategy of multipartite viruses. Annu Rev Virol. 2020;7:203–218. doi: 10.1146/annurev-virology-010220-063346 . 32991271

[45] Sicard A, Pirolles E, Gallet R, Vernerey MS, Yvon M, Urbino C, et al. A multicellular way of life for a multipartite virus. eLife. 2019;8:e43599. doi: 10.7554/eLife.43599. 30857590 PMC6414197

[46] Sicard A, Michalakis Y, Gutierrez S, Blanc S. The strange lifestyle of multipartite viruses. PLoS Pathog. 2016;12:e1005819. doi: 10.1371/journal.ppat.1005819. 27812219 PMC5094692

[47] Li C, Shi M, Tian J, Lin X, Kang Y, Chen L, et al. Unprecedented genomic diversity of RNA viruses in arthropods reveals the ancestry of negative-sense RNA viruses. eLife. 2015;4:eo5378. doi: 10.7554/eLife.05378. 25633976 PMC4384744

[48] Ladner JT, Wiley MR, Beitzel B, Auguste AJ, Dupuis APn, Lindquist ME, et al. A multicomponent animal virus isolated from mosquitoes. Cell Host Microbe. 2016;20:357–367. doi: 10.1016/j.chom.2016.07.011 . 27569558 PMC5025392

[49] Zhang X, Wang N, Wang Z, Liu Q. The discovery of segmented flaviviruses: implications for viral emergence. Curr Opin Virol. 2020;40:11–18. doi: 10.1016/j.coviro.2020.02.001 . 32217446

[50] Zwart MP, Elena SF. Modeling multipartite virus evolution: the genome formula facilitates rapid adaptation to heterogeneous environments(dagger). Virus Evol. 2020;6:veaa022. doi: 10.1093/ve/veaa022. 32405432 PMC7206449

[51] Hamid MR, Xie J, Wu S, Maria SK, Zheng D, Assane Hamidou A, et al. A novel deltaflexivirus that infects the plant fungal pathogen, *Sclerotinia sclerotiorum*, can be transmitted among host vegetative incompatible strains. Viruses. 2018;10:295. doi: 10.3390/v10060295. 29857477 PMC6024712

[52] Singleton MR, Dillingham MS, Wigley DB. Structure and mechanism of helicases and nucleic acid translocases. Annu Rev Biochem. 2007;76:23–50. doi: 10.1146/annurev.biochem.76.052305.115300 . 17506634

[53] Koonin EV, Dolja VV. Evolution and taxonomy of positive-strand RNA viruses:implications of comparative analysis of amino acid sequences. Crit Rev Biochem Mol Biol. 1993;28:375–430. doi: 10.3109/10409239309078440 . 8269709

[54] Quito-Avila DF, Brannen PM, Cline WO, Harmon PF, Martin RR. Genetic characterization of Blueberry necrotic ring blotch virus, a novel RNA virus with unique genetic features. J Gen Virol. 2013;94:1426–1434. doi: 10.1099/vir.0.050393-0 . 23486668

[55] Morozov SY, Solovyev AG. Phylogenetic relationship of some "accessory" helicases of plant positive-stranded RNA viruses: toward understanding the evolution of triple gene block. Front Microbiol. 2015;6:508. doi: 10.3389/fmicb.2015.00508. 26042118 PMC4436898

[56] Tuomivirta TT, Kaitera J, Hantula J. A novel putative virus of *Gremmeniella abietina type* B (Ascomycota: Helotiaceae) has a composite genome with endornavirus affinities. J Gen Virol. 2009;90:2299–2305. doi: 10.1099/vir.0.011973-0 . 19494051

[57] Picarelli MASC, Forgia M, Rivas EB, Nerva L, Chiapello M, Turina M, et al. Extreme diversity of mycoviruses present in isolates of *Rhizoctonia solani* AG2-2 LP from *Zoysia japonica* from Brazil. Front Cell Infect Microbiol. 2019;9:244. doi: 10.3389/fcimb.2019.00244. 31355150 PMC6640214

[58] Tomlinson JA, Garrett RG. Role of *Olpidium* in the transmission of big vein disease of lettuce. Nature. 1962;194:249–250. doi: 10.1038/194249a0

[59] Campbell RN. Relationship between the lettuce big-vein virus and its vector, *Olpidium Brassicae*. Nature. 1962;195:675–677. doi: 10.1038/195675a0 13876064

[60] Valverde RA, Khalifa ME, Okada R, Fukuhara T, Sabanadzovic S, ICTV Report C. ICTV virus taxonomy profile: *Endornaviridae*. J Gen Virol. 2019;100:1204–1205. doi: 10.1099/jgv.0.001277 . 31184570 PMC12643110

[61] Xie J, Wei D, Jiang D, Fu Y, Li G, Ghabrial S, et al. Characterization of debilitation-associated mycovirus infecting the plant-pathogenic fungus *Sclerotinia sclerotiorum*. J Gen Virol. 2006;87:241–249. doi: 10.1099/vir.0.81522-0 . 16361437

[62] Liu L, Cheng J, Fu Y, Liu H, Jiang D, Xie J. New insights into reovirus evolution: implications from a newly characterized mycoreovirus. J Gen Virol. 2017;98:1132–1141. doi: 10.1099/jgv.0.000752 . 28548042

[63] Mu F, Xie J, Cheng S, You MP, Barbetti MJ, Jia J, et al. Virome characterization of a collection of *Sclerotinia sclerotiorum* from Australia. Front Microbiol. 2018;8:2540. doi: 10.3389/fmicb.2017.02540. 29375495 PMC5768646

[64] Wang Q, Cheng S, Xiao X, Cheng J, Fu Y, Chen T, et al. Discovery of two mycoviruses by high-throughput sequencing and assembly of mycovirus-derived small silencing RNAs from a hypovirulent strain of *Sclerotinia sclerotiorum*. Front Microbiol. 2019;10:1415. doi: 10.3389/fmicb.2019.01415 WOS:000473569200001. 31338072 PMC6626909

[65] Bolger AM, Lohse M, Usadel B. Trimmomatic: a flexible trimmer for Illumina sequence data. Bioinformatics. 2014;30:2114–2120. doi: 10.1093/bioinformatics/btu170 . 24695404 PMC4103590

[66] Kim D, Langmead B, Salzberg SL. HISAT: a fast spliced aligner with low memory requirements. Nat Methods. 2015;12:357–360. doi: 10.1038/nmeth.3317 . 25751142 PMC4655817

[67] Bankevich A, Nurk S, Antipov D, Gurevich AA, Dvorkin M, Kulikov AS, et al. SPAdes: a new genome assembly algorithm and its applications to single-cell sequencing. J Comput Biol. 2012;19:455–477. doi: 10.1089/cmb.2012.0021 . 22506599 PMC3342519

[68] Potgieter AC, Page NA, Liebenberg J, Wright IM, Landt O, van Dijk AA. Improved strategies for sequence-independent amplification and sequencing of viral double-stranded RNA genomes. J Gen Virol. 2009;90:1423–1432. doi: 10.1099/vir.0.009381-0 . 19264638

[69] Scotto-Lavino E, Du G, Frohman MA. 5’ end cDNA amplification using classic RACE. Nat Protoc. 2006;1:2555–2562. doi: 10.1038/nprot.2006.480 . 17406509

[70] Katoh K, Kuma K, Toh H, Miyata T. MAFFT version 5: improvement in accuracy of multiple sequence alignment. Nucleic Acids Res. 2005;33:511–518. doi: 10.1093/nar/gki198 . 15661851 PMC548345

[71] Capella-Gutierrez S, Silla-Martinez JM, Gabaldon T. trimAl: a tool for automated alignment trimming in large-scale phylogenetic analyses. Bioinformatics. 2009;25:1972–1973. doi: 10.1093/bioinformatics/btp348 . 19505945 PMC2712344

[72] Guindon S, Dufayard JF, Lefort V, Anisimova M, Hordijk W, Gascuel O. New algorithms and methods to estimate maximum-likelihood phylogenies: assessing the performance of PhyML 3.0. Syst Biol. 2010;59:307–321. doi: 10.1093/sysbio/syq010 . 20525638

[73] Zhang L, Fu Y, Xie J, Jiang D, Li G, Yi X. A novel virus that infecting hypovirulent strain XG36-1 of plant fungal pathogen *Sclerotinia sclerotiorum*. Virol J. 2009;6:96. doi: 10.1186/1743-422X-6-96. 19583873 PMC2714488

[74] Jia H, Dong K, Zhou L, Wang G, Hong N, Jiang D, et al. A dsRNA virus with filamentous viral particles. Nat Commun. 2017;8:168. doi: 10.1038/s41467-017-00237-9. 28761042 PMC5537263

[75] Wang Q, Mu F, Xie J, Cheng J, Fu Y, Jiang D. A single ssRNA segment encoding RdRp is sufficient for replication, infection, and transmission of ourmia-like virus in fungi. Front Microbiol. 2020;11:379. doi: 10.3389/fmicb.2020.00379. 32256466 PMC7093599

