# Supplementary figures and images for: Nine viruses from eight lineages exhibiting new evolutionary modes that co-infect a hypovirulent phytopathogenic fungus
Source: PLoS Pathog. 2021 Aug 24;17(8):e1009823. doi: 10.1371/journal.ppat.1009823 (PMC8415603; doi:10.1371/journal.ppat.1009823)

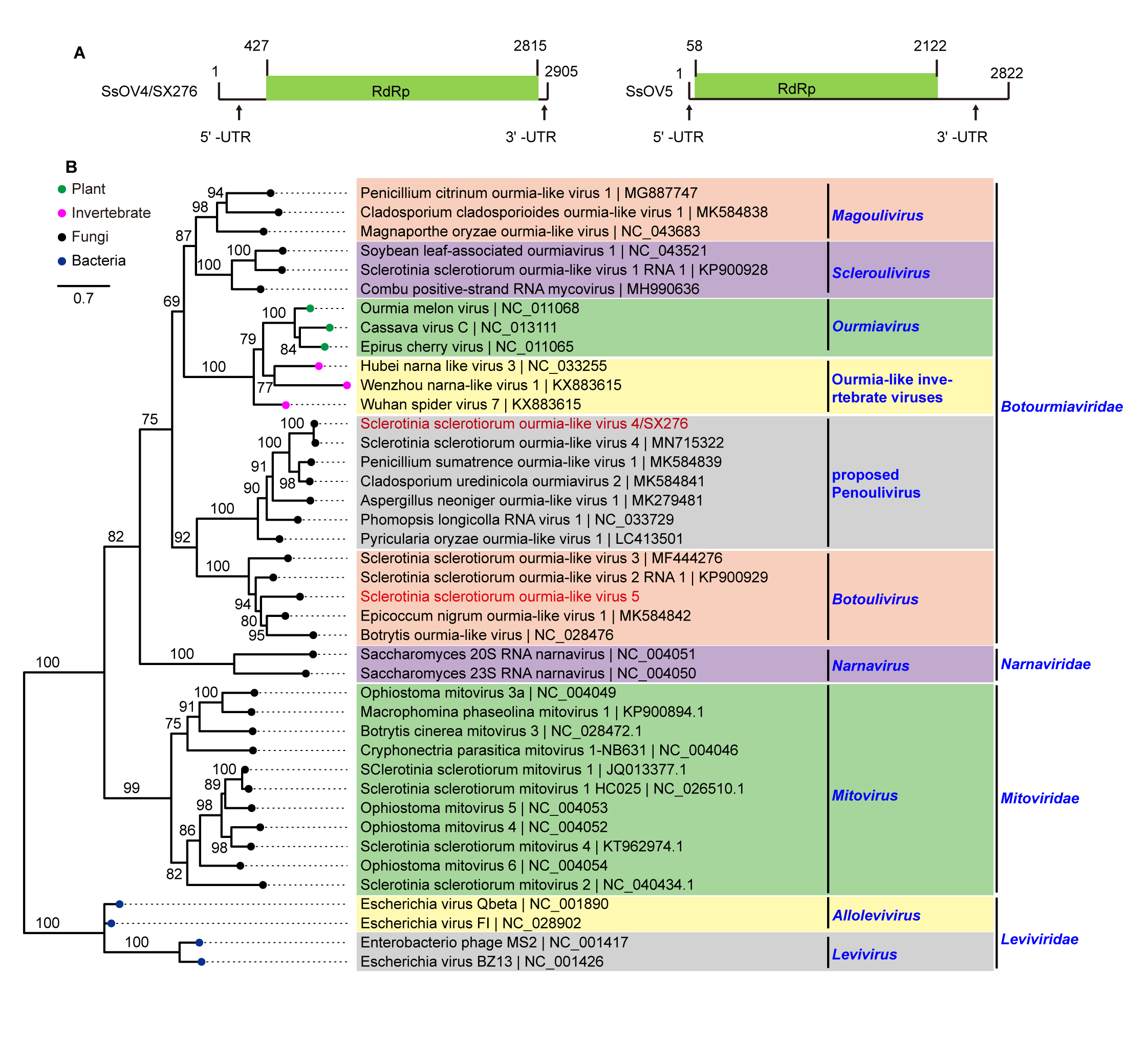

Supplement: S1 Fig — (A) Schematic organization and annotations of SsOV4/SX276 and SsOV5 genome. Open reading frames (ORFs) are shown as boxes, the conserved motifs were represented in different colors. (B) Phylogenetic analysis of SsOV4/SX276 and SsOV5. A maximum-likelihood phylogenetic tree was constructed based on amino acid alignments of RdRp. Bootstrap values (%) obtained with 1000 replicates are indicated on the branches, and branch lengths correspond to genetic distance; the scale bar at the lower left corresponds to genetic distance. The novel viruses of SsOV4/SX276 and SsOV5 are highlighted with red font. (TIF) [file ppat.1009823.s001.tif]

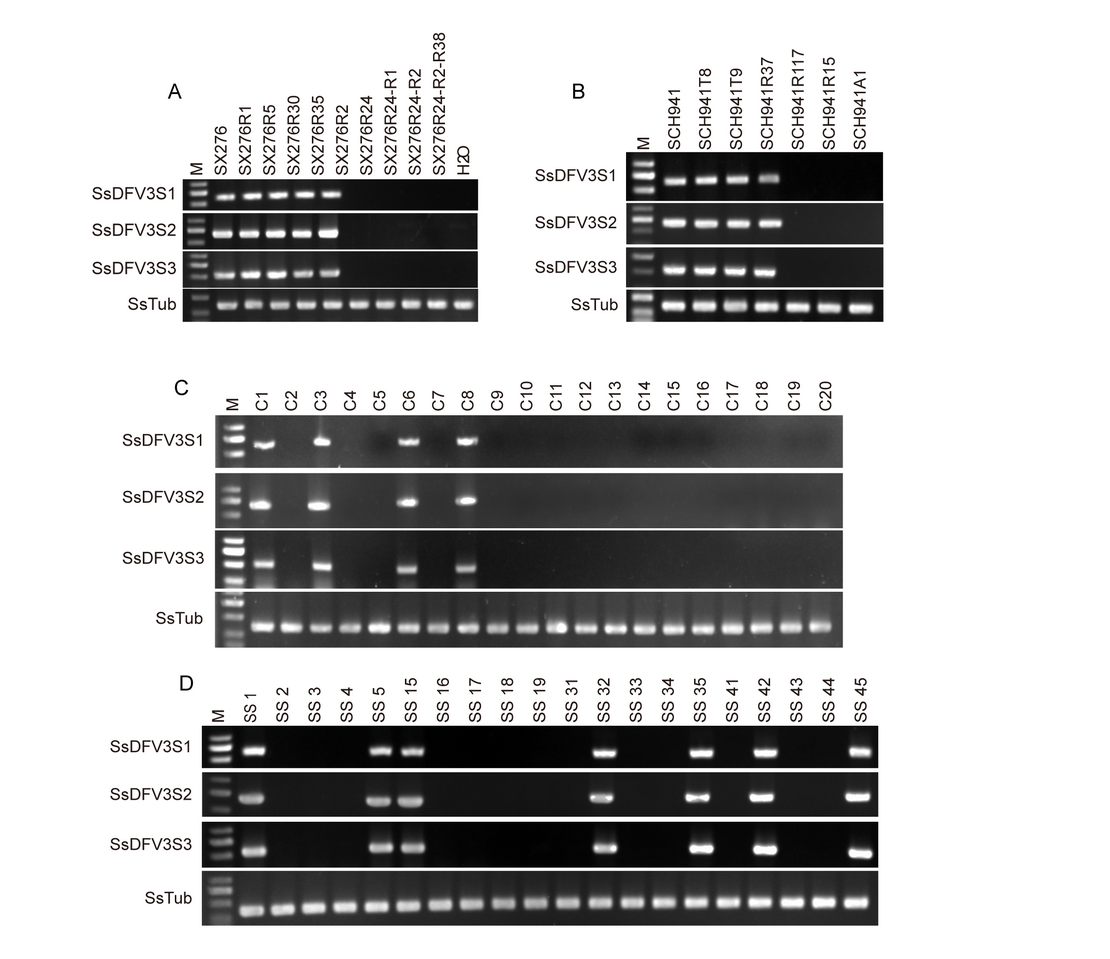

Supplement: S2 Fig — (A) Detection of SsDFV3S1-S3 in protoplast regeneration strains of SX276. (B) Detection of SsDFV3S1-S3 in protoplast regeneration strains of SCH941. (C) RT-PCR was carried out to determine the group of segments S1-S3 among 100 isolates of S. sclerotinia from a single crop field collected in May 2017. C1-C20 represent twenty groups, and each group contains five strains. (D) RT-PCR was performed to determine the strains of SsDFV3S1-S3. (TIF) [file ppat.1009823.s002.tif]

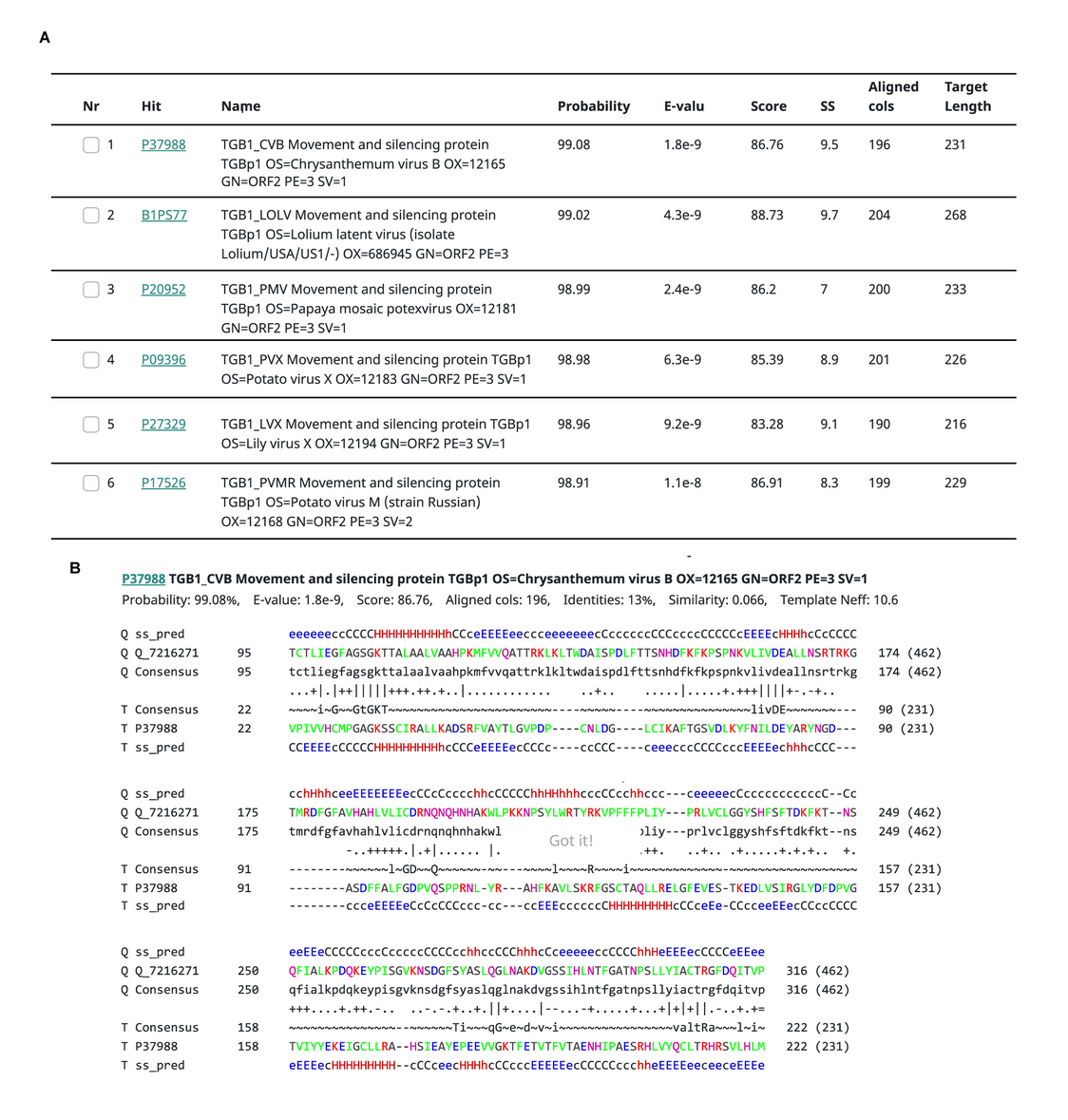

Supplement: S3 Fig — (A) The 5 most similar viruses with the Hel-2 of SsDFV3 in Protein Data Bank (PDB). (B) Sequence alignment of the Hel-2 domain from SsDFV3 and Chrysanthemum virus B. (TIF) [file ppat.1009823.s003.tif]

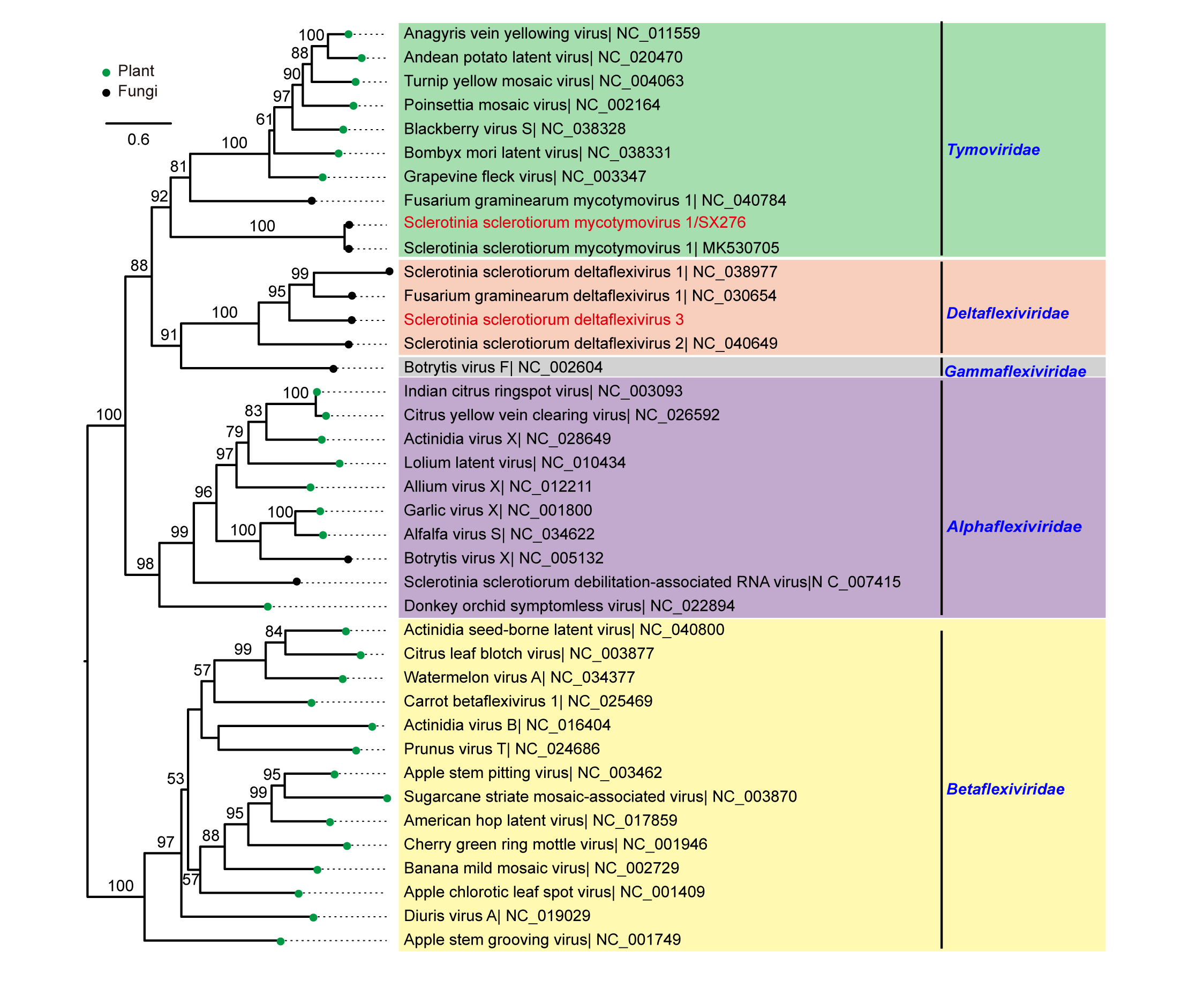

Supplement: S4 Fig — Phylogenetic analysis of SsMTV1/SX276 and SsDFV3 constructed based on amino acid alignments of viral methyltransferase sequence. The tree was constructed by a maximum likelihood method. The novel viruses SsMTV1/SX276 and SsDFV3 are highlighted with red font. (TIF) [file ppat.1009823.s004.tif]

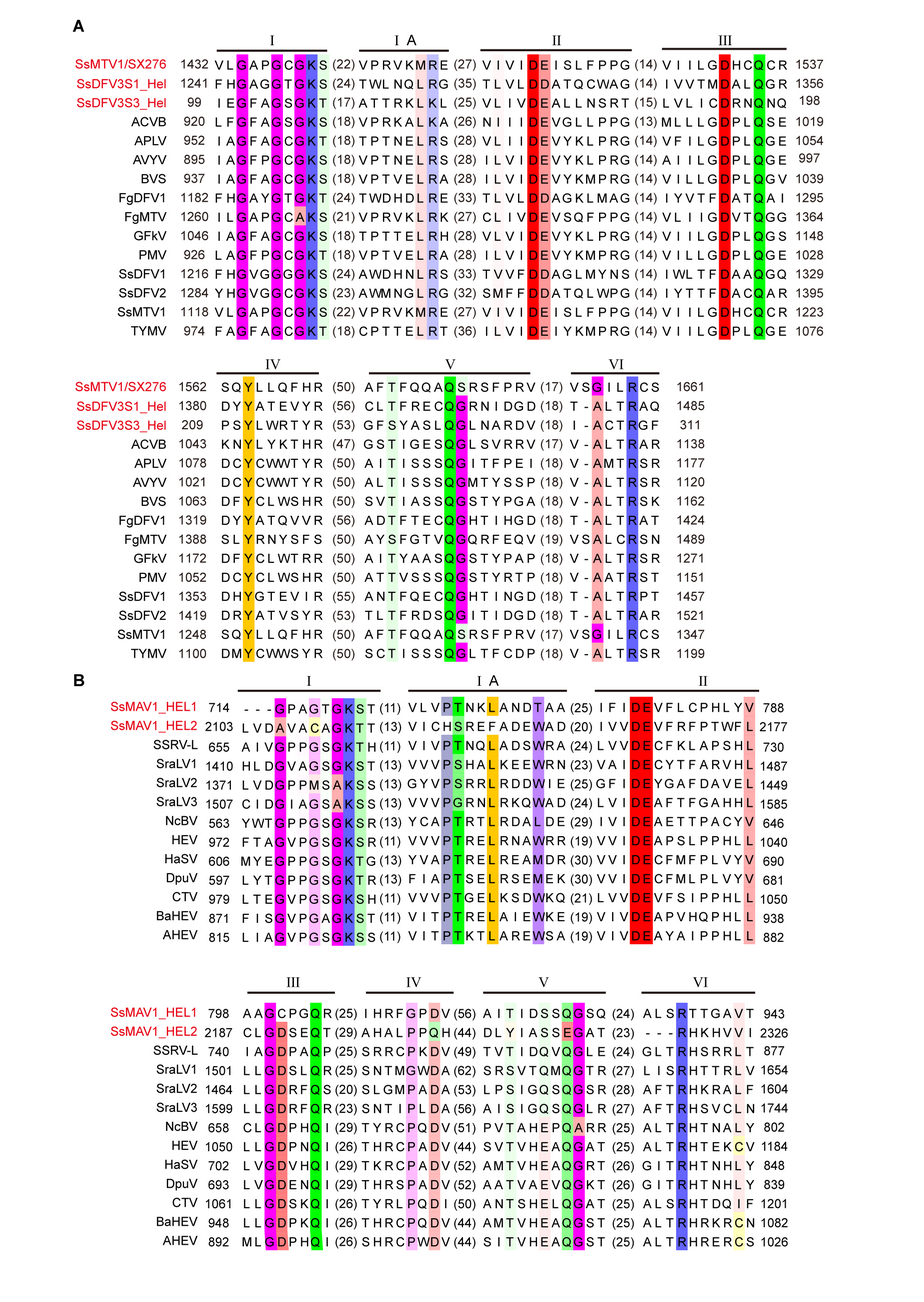

Supplement: S5 Fig — (A) Multiple alignments based on the helicase amino acid sequence of SsMTV1/SX276 and SsDFV3 and other viruses in order Tymovirales. (B) Multiple alignments based on the helicase amino acid sequence of SsMAV1 and other viruses in alphavirus sub-group. (TIF) [file ppat.1009823.s005.tif]

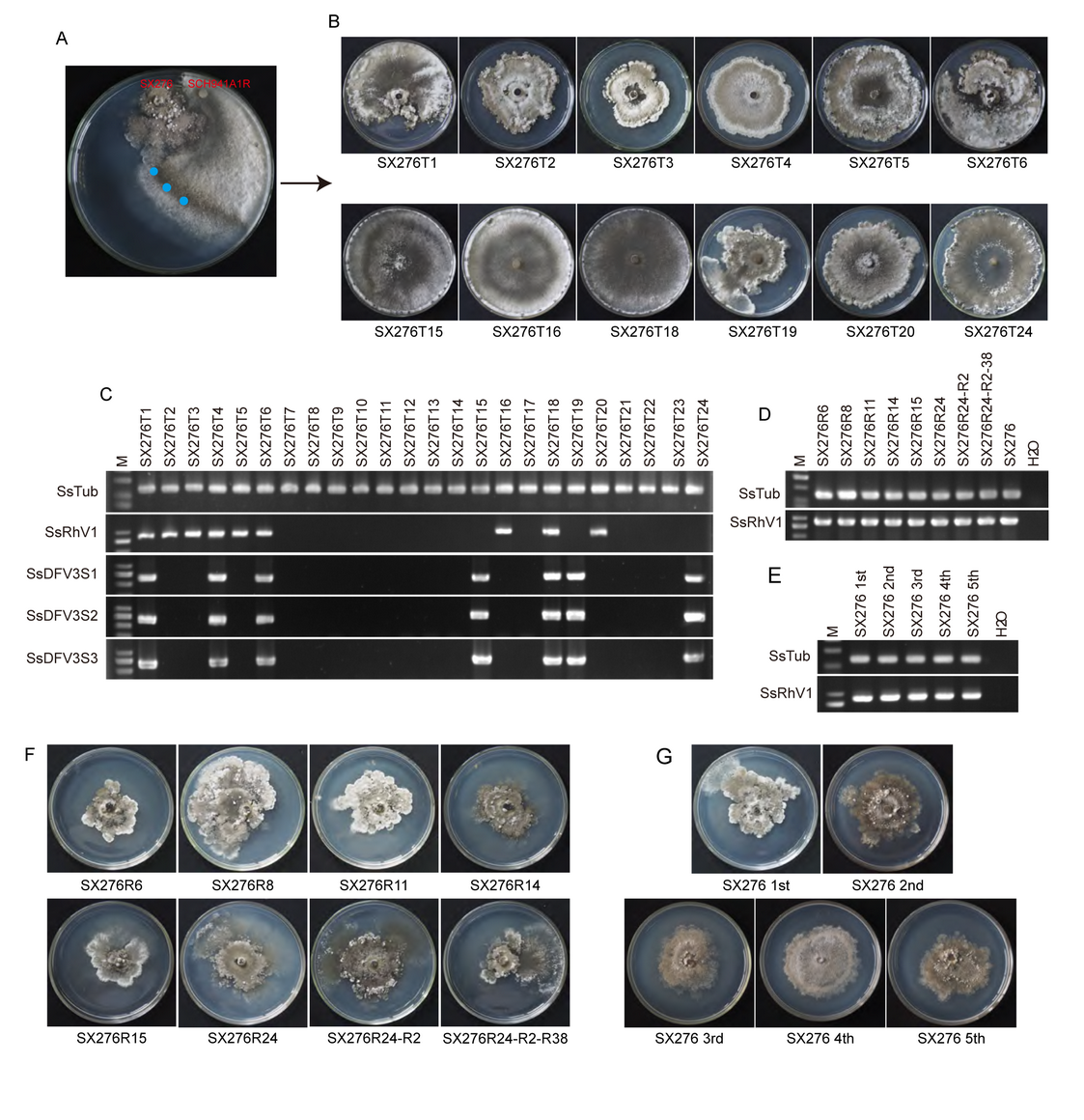

Supplement: S6 Fig — (A) Co-culture of strain SX276 and SCH941A1R (15 dpi). The new isolates (indicated by blue circles) were picked up from the colony margin of the virulent strain. (B) The colony morphology of the virus recipient strains (7 dpi). (C) The detection of the SsRhV1 and SsDFV3 in the virus recipient strains. (D) RT-PCR was performed to detect the SsRhV1 in the protoplast isolations from strain SX276. (E) Detection of SsRhV1 was performed during strain SX276 subculturing. (F) Colony morphology of the protoplast isolations from strain SX276 (7 dpi). (G) Colony morphology of the strain SX276 after subculturing 1 to 5 times. (TIF) [file ppat.1009823.s006.tif]

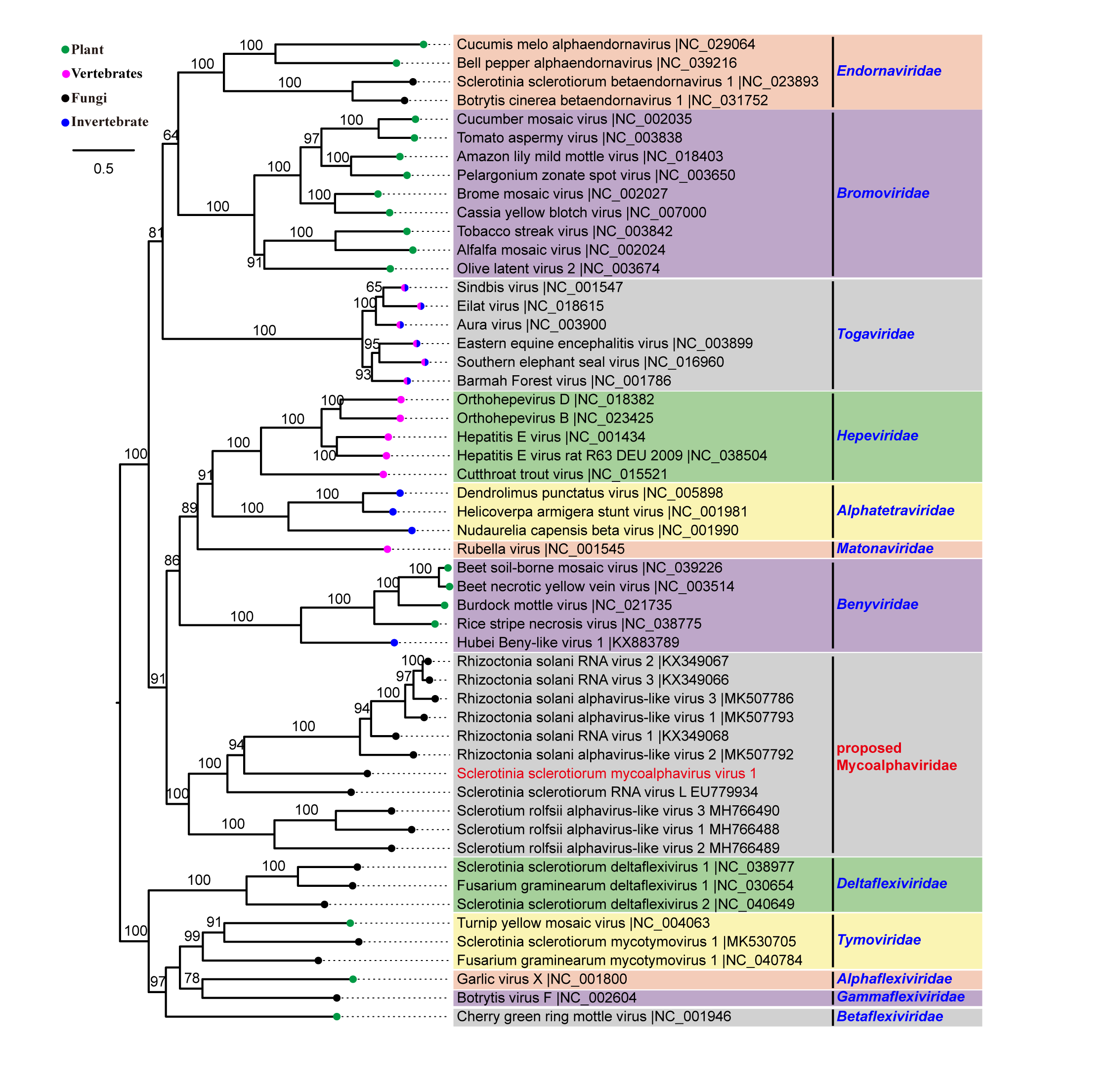

Supplement: S7 Fig — Phylogenetic analysis of SsMAV1. A maximum-likelihood phylogenetic tree was constructed based on amino acid alignments of RdRp. The novel virus of SsMAV1 is highlighted in red font. (TIF) [file ppat.1009823.s007.tif]

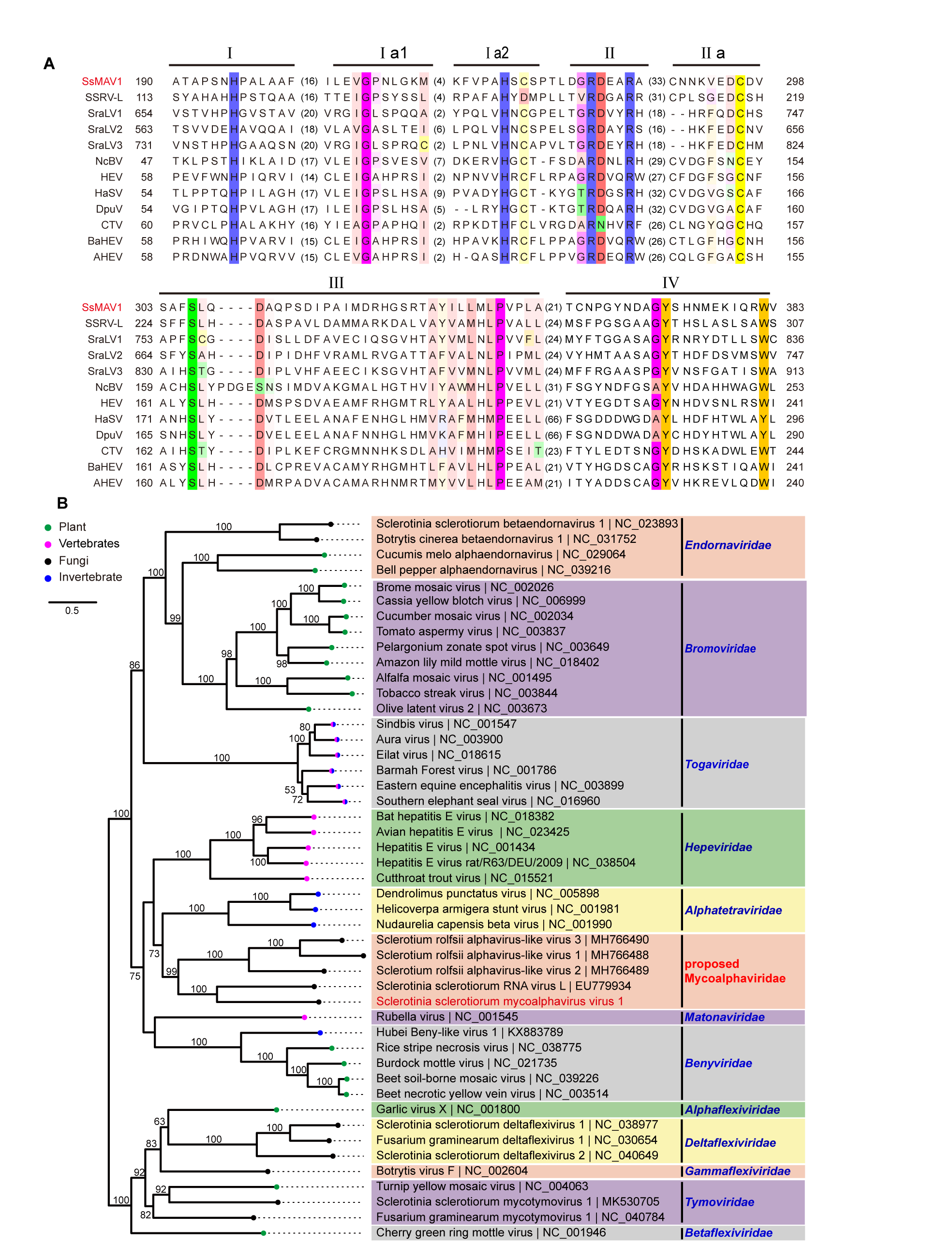

Supplement: S8 Fig — (A) Multiple alignments based on the methyltransferase amino acid sequence of SsMAV1 and other viruses in alphavirus sub-group. (B) Phylogenetic analysis of SsMAV1 constructed based on amino acid alignments of viral methyltransferase sequence. The tree was constructed by a maximum likelihood method. SsMAV1 is highlighted in red font. (TIF) [file ppat.1009823.s008.tif]

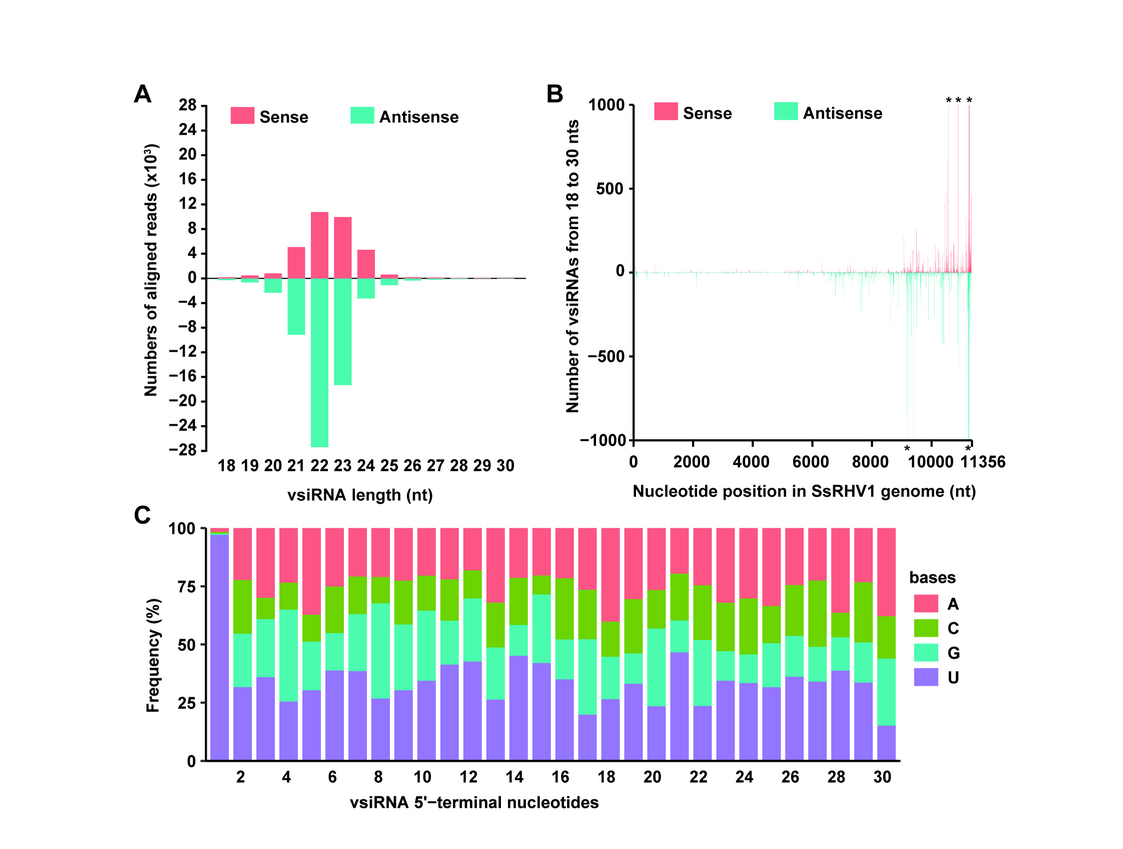

Supplement: S9 Fig — (A) Size distribution of vsiRNAs derived from SsRhV1. x-axis represents the size of vsiRNAs, and the y-axis shows numbers; red represents vsiRNAs derived from sense, and the cyan-blue represents vsiRNAs derived from anti-sense. (B) Distribution of the vsiRNAs mapped on the corresponding genome of SsRhV1. The x-axis shows schematic genomic organization of SsRhV1. The y-axis indicates the numbers of vsiRNAs matching the sense strand (red, above the x-axis) and antisense strand (cyan-blue, below the x-axis). (C) The relative frequency of 5’-terminal nucleotide of vsiRNAs. The x-axis represents the vsiRNAs size (nt), and the y-axis shows the percentages of 5’-terminal nucleotides consisting of G, C, A, and U in the 18- to 30-nt vsiRNAs class. (TIF) [file ppat.1009823.s009.tif]

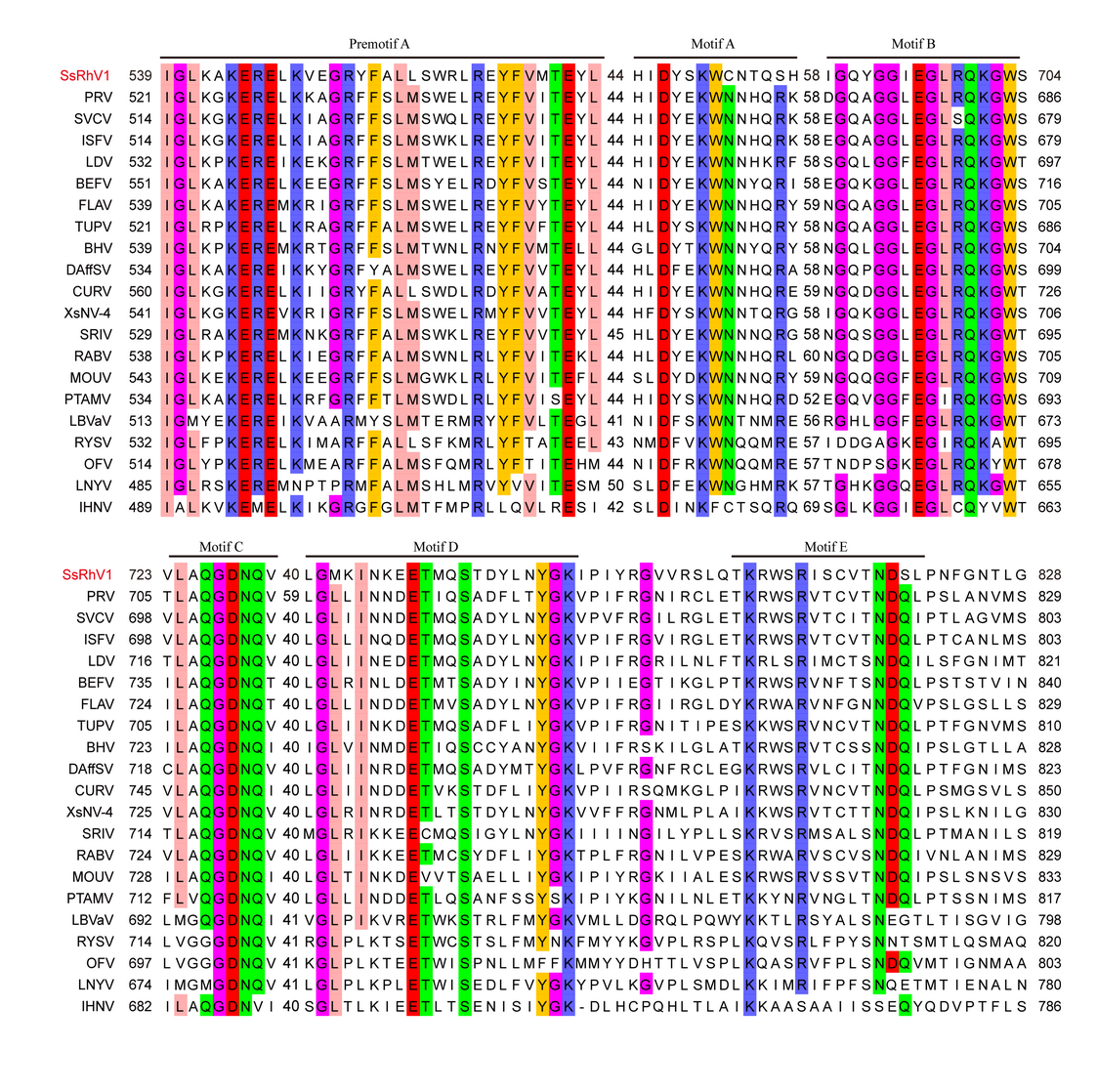

Supplement: S10 Fig — Multiple alignments based on the RdRp amino acid sequence of SsRhV1 and other viruses in family Rhabdoviridae. (TIF) [file ppat.1009823.s010.tif]

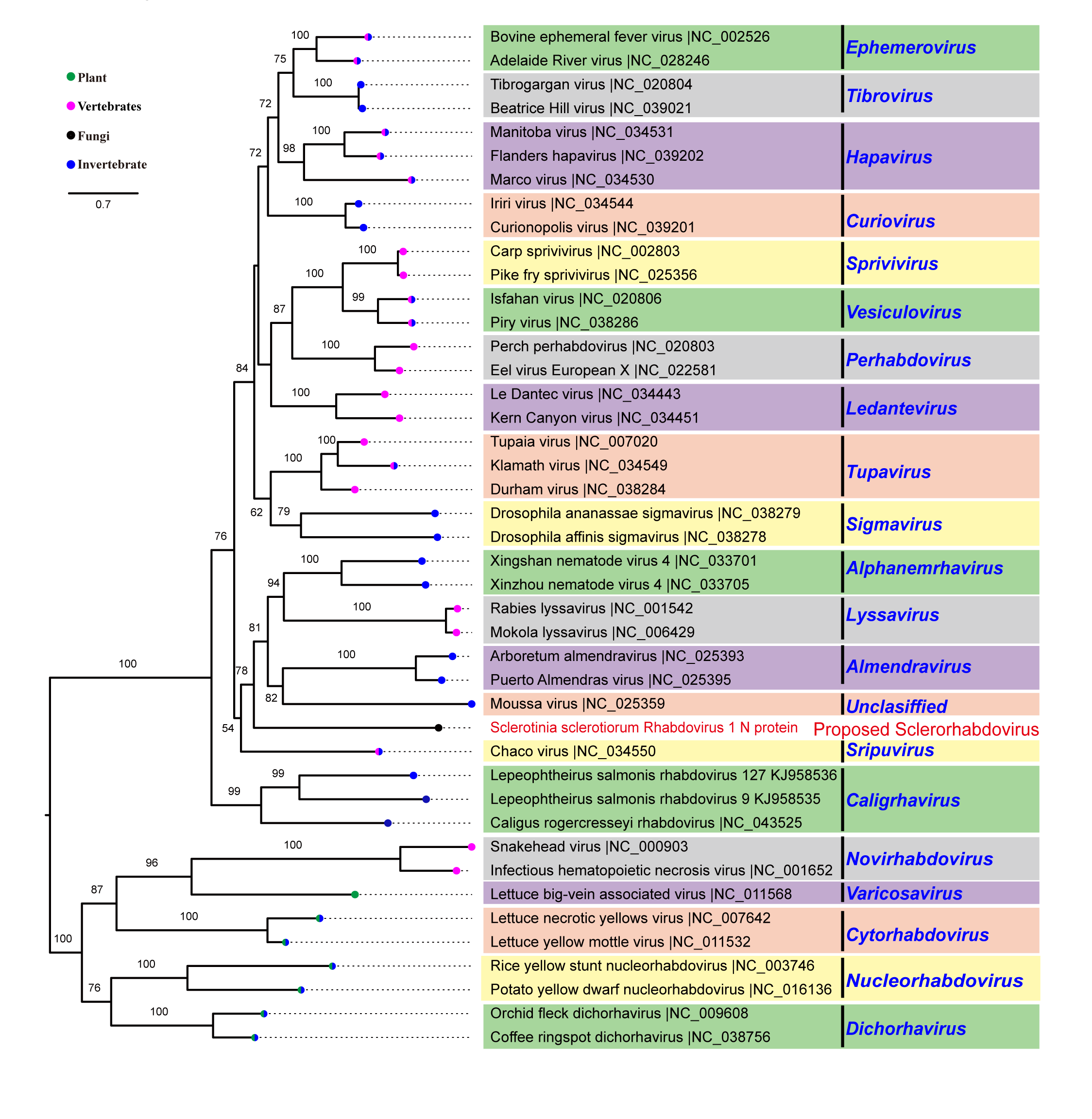

Supplement: S11 Fig — The tree was constructed by a maximum likelihood method. The virus SsRhV1 is represented by the red font. (TIF) [file ppat.1009823.s011.tif]

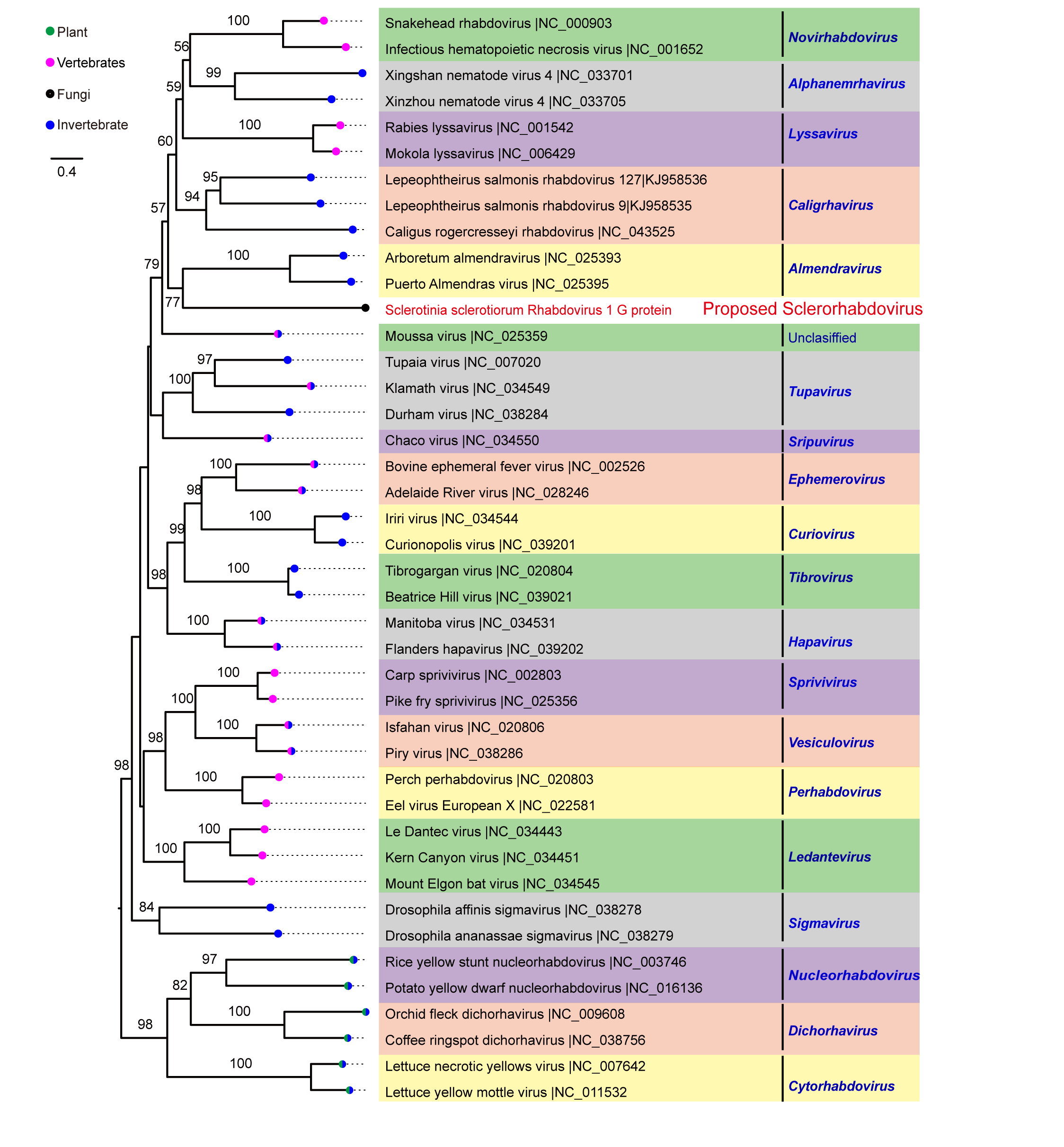

Supplement: S12 Fig — The tree was constructed by a maximum likelihood method. The virus SsRhV1 is represented by the red font. (TIF) [file ppat.1009823.s012.tif]

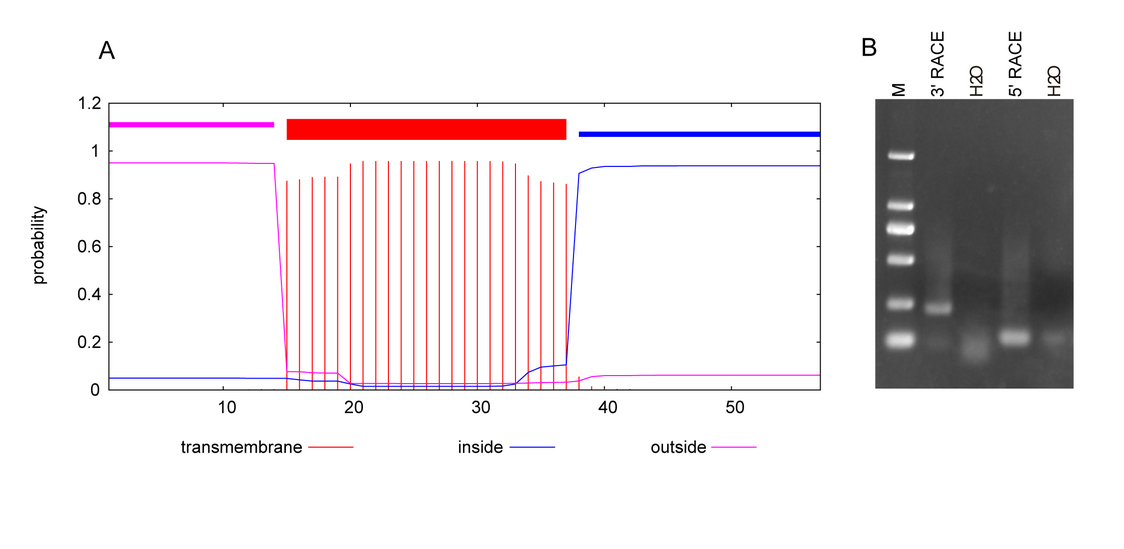

Supplement: S13 Fig — (A) The transmembrane domain prediction of G1 protein. (B) Agarose gel electrophoresis of the 3′ and 5′ RACE of G1 ORF on 1% agarose gel. (TIF) [file ppat.1009823.s013.tif]

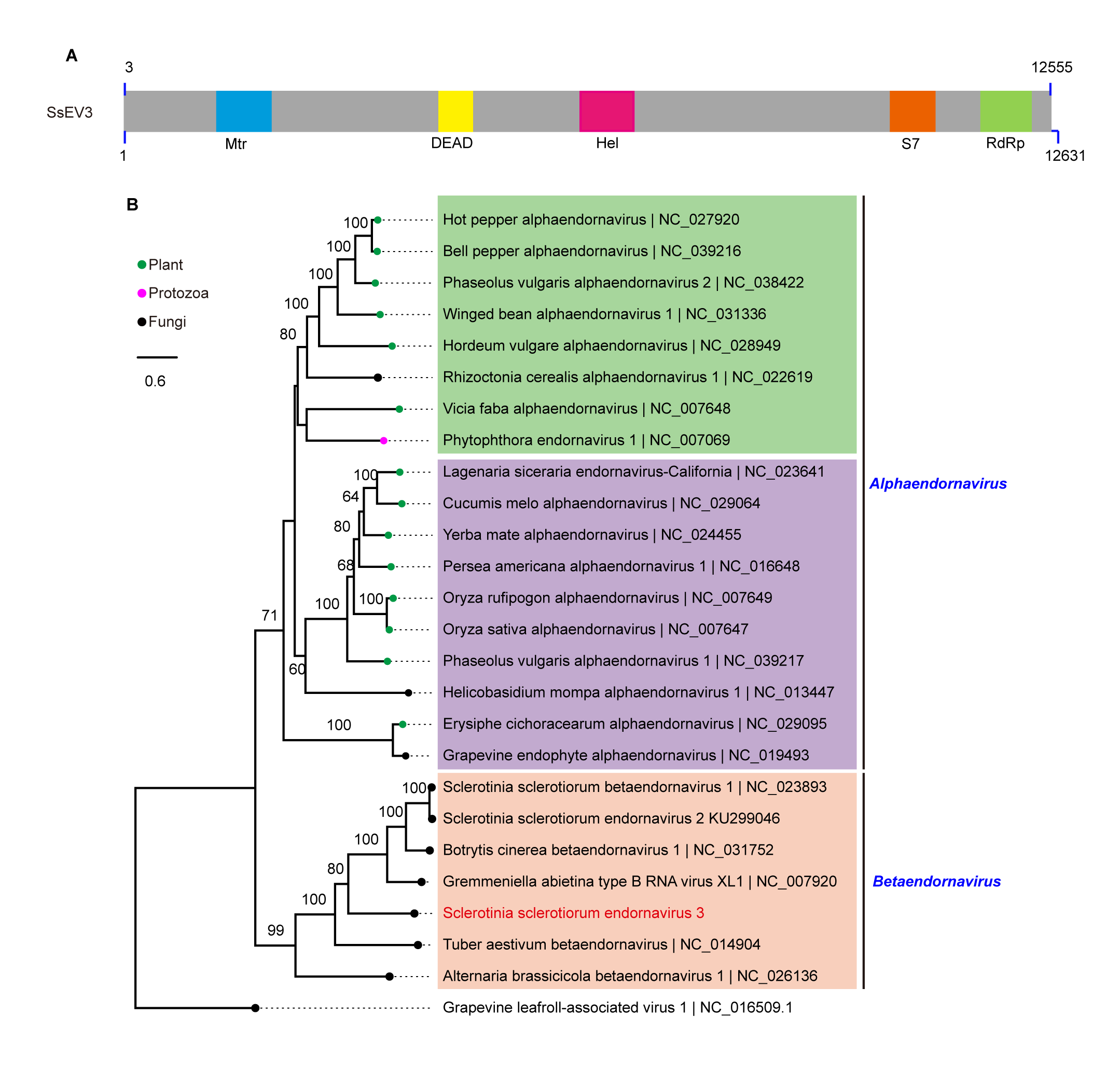

Supplement: S14 Fig — (A) The organizations of SsEV3. Open reading frames (ORFs) are shown as boxes. (B) Phylogenetic analysis of SsEV3. A maximum-likelihood phylogenetic tree was constructed based on amino acid alignments of RdRp. SsEV3 is highlighted in red font. (TIF) [file ppat.1009823.s014.tif]

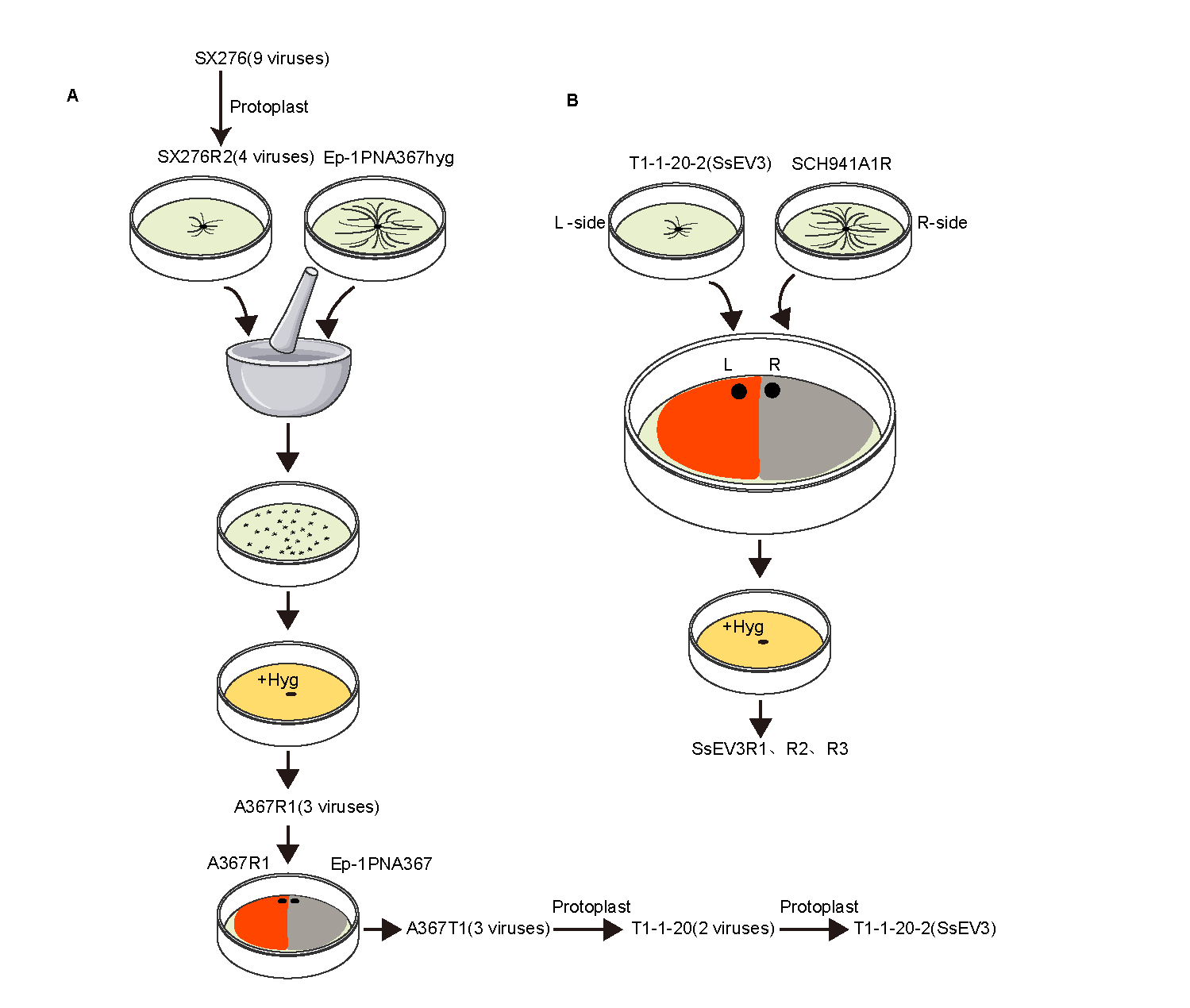

Supplement: S15 Fig — (A) The procedure to the strain T1-1-20-2 only infected with SsEV3 through protoplast regeneration and dual-culture. (B) Dual-culture of strains T1-1-20-2 and SCH941A1R. (TIF) [file ppat.1009823.s015.tif]

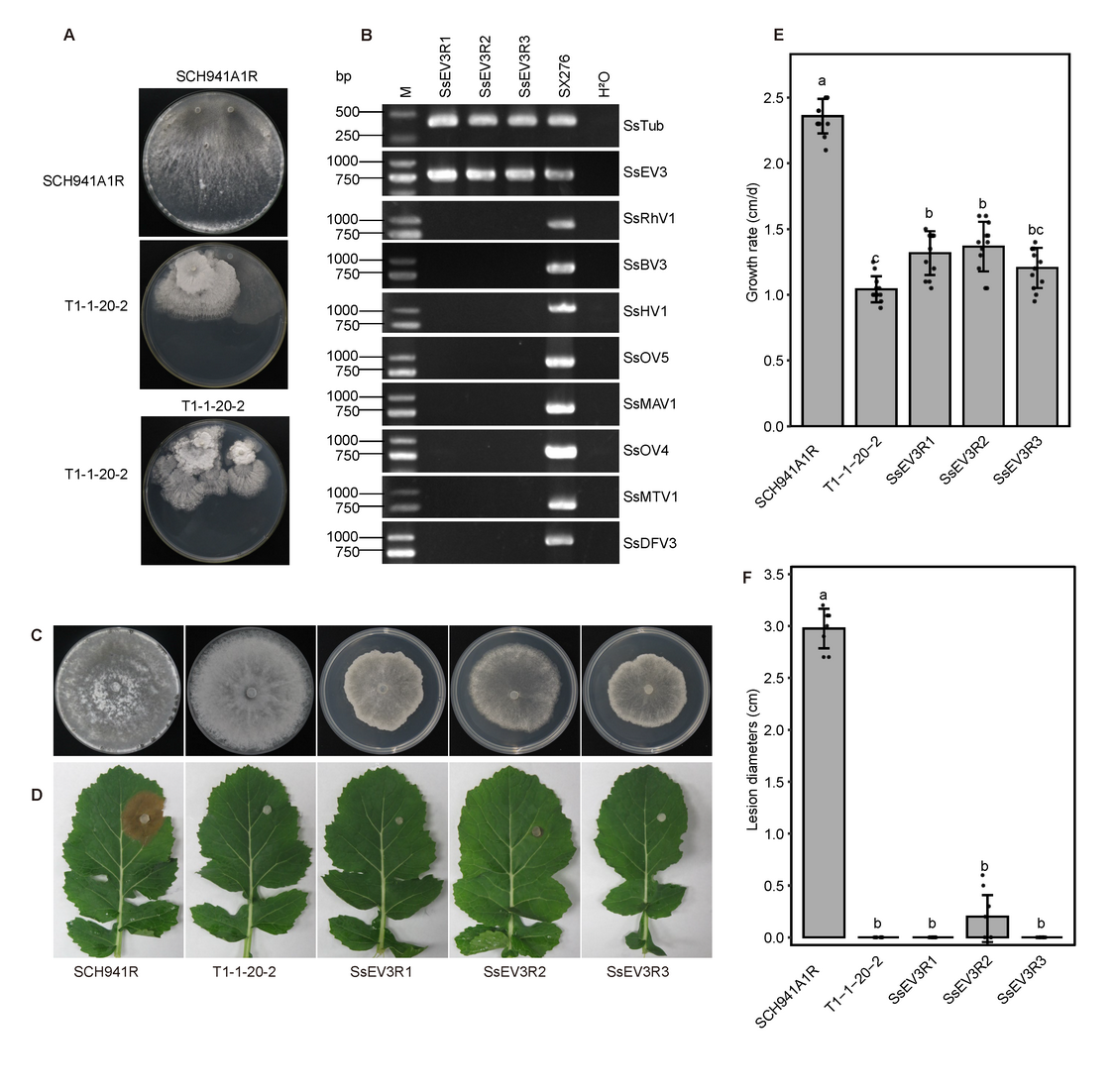

Supplement: S16 Fig — (A) Dual-culture of strains T1-1-20-2 and SCH941A1R (10 dpi), dual-culture of two SCH941A1R strains were as the control. (B) The detection of the viruses in the virus recipient strains. (C) The colony morphology of the individual strains (5 dpi). (D)The determination of pathogenicity of the individual strains. (E) and (F) the growth rate and diameter of leave lesion (48 hpi). Error bars indicate the SD from six sample means. The different letters on the top of each column indicate significantly difference at the P < 0.05 level of confidence according to the t-test. (TIF) [file ppat.1009823.s016.tif]
